# Association of ABO blood groups with ovarian reserve, and outcomes after assisted reproductive technology: systematic review and meta-analyses

**DOI:** 10.1186/s12958-020-00685-x

**Published:** 2021-02-06

**Authors:** Jing Zhao, Zhongyuan Yao, Jie Hao, Bin Xu, Yonggang Wang, Yanping Li

**Affiliations:** 1 Reproductive Medicine Center, Xiangya Hospital, Central South University, 87 Xiangya Road, Changsha City, Hunan Province People’s Republic of China; 2 Clinical Research Center For Women’s Reproductive Health In Hunan Province, Changsha, Hunan China

**Keywords:** ABO blood groups; assisted reproductive technology (ART), Diminished ovarian reserve (DOR), Ovarian hyperstimulation syndrome (OHSS), Live birth

## Abstract

**Background:**

There has been an interest in the relationship between ABO blood groups and infertility. Many studies have investigated the association of ABO blood groups with diminished ovarian reserve (DOR), ovarian hyperstimulation syndrome (OHSS), and outcomes of assisted reproductive technology (ART), with controversial results.

**Methods:**

A systematic review and meta-analysis was conducted to evaluating the association of ABO blood groups with DOR, OHSS, and outcomes of ART.

**Results:**

Thirteen studies performed between 2010 and 2018 were included in this meta-analysis. DOR, OHSS, live birth rate (LBR), clinical pregnancy rate (CPR), miscarriage rate (MR) were reported in 9, 2, 4, 3, 2 studies, respectively. The combined results showed similar risk of DOR among individuals with blood group A (RR, 0.98; 95% confidence interval [CI], 0.85, 1.13), B (RR, 0.96; 95% CI, 0.76, 1.20), AB (RR, 1.00; 95% CI, 0.76, 1.30), and non-O (RR, 0.94; 95% CI, 0.79, 1.11) as compared to those with blood group O. Meta-analysis showed that the incidences of OHSS were similar in women with blood group A (RR, 1.05; 95% CI, 0.66, 1.66), B (RR, 1.04; 95% CI, 0.46, 2.35), AB (RR, 0.51; 95% CI, 0.10, 2.56), non-O (RR, 1.02; 95% CI, 0.65, 1.57) with blood group O. As to the clinical outcomes, meta-analysis showed no difference in LBR among individuals with blood group A (RR, 1.27; 95% CI, 0.74, 2.17), B (RR, 1.47; 95% CI, 0.95, 2.29), AB (RR, 1.48; 95% CI, 0.76, 2.90), non-O (RR, 1.28; 95% CI, 0.83, 1.98) when compared to those with blood group O. Similarly, the results also found that there were no difference in CPR and MR between women with blood A (CPR: RR, 1.12), B (CPR: RR, 1.08), AB (CPR: RR, 1.05), non-O (CPR: RR, 1.05; MR: RR, 0.94) and blood group O.

**Conclusions:**

ABO blood groups may not be associated with DOR, OHSS, LBR, CPR, and MR of ART. Infertility and ART outcomes are influenced by multiple factors. Blood groups should not be taken into account excessively during diagnosis and treatment of infertile women.

**Supplementary Information:**

The online version contains supplementary material available at 10.1186/s12958-020-00685-x.

## Introduction

The ABO blood group system is a representation of the human blood group antigens expressed on the surface of red blood cells. The ABO blood group antigen plays an important role in immunology and organ transplantation [1]. Recently, studies have reported that ABO blood group was associated with the some gynecological diseases, such as endometriosis [2] and ovarian cancer [3].

Early in the 1960s, there was study that suggested that ABO blood group incompatibility might be an immune factor of infertility [4]. Many studies have reported a high risk of thrombosis in people with non-O blood group [5] [6]. Fifty years have passed, and some studies have explored the relationship between the ABO blood group and female infertility, including DOR, OHSS, RSA, etc. But unfortunately, the conclusions were inconsistent.

One study has observed the relationship between blood group and IVF-ET pregnancy outcomes and found that blood group B is associated with increased live birth rate [7]. However, subsequent studies have confirmed that ABO blood group is not related to the IVF pregnancy outcome [1, 8]. Some studies have also reported the relationship between the ABO blood group and ovarian reserve, and showed that blood group O is more likely to have diminished ovarian reserve (DOR) [9, 10]. Whereas some studies found that women with antigen B are more likely to have DOR [11], and subsequent studies found that ABO blood group was not related to ovarian reserve or ovarian response [12–17]. Research reports on the relationship between ABO blood group and ovarian hyperstimulation syndrome (OHSS) were also inconsistent [18, 19]. Binder found that blood group A is related to the occurrence of early-onset OHSS in IVF, but other studies did not showed a relationship between ABO blood group and early or late OHSS [20].

Although so many clinical trials have studied the relationship between ABO blood group and ovarian reserve, OHSS, and IVF outcomes, there is no systematic review and meta-analysis on this issue.

This study aimed to evaluate the association of ABO blood group and ovarian reserve, OHSS, and pregnancy outcomes of ART-treated women through a systematic review and meta-analysis of existing literature.

## Methods

### Search strategy

A systematic search of all published studies was performed using the PubMed, EMBASE, and Google Scholar from 1966 to April 2020. The key words used for the search were as follows: a term including blood group (ABO blood group, ABO blood type), a term included the infertility (diminished ovarian reserve, DOR, ovarian hyperstimulation syndrome, OHSS, assisted reproductive technology, ART, clinical pregnancy, live birth, miscarriage). These subsets were combined with “AND” to produce literatures related to our research subject. The cohort, retrospective, or prospective studies published in English was included in our study. Two authors independently reviewed the included literature. If there was a disagreement, the third author resolved it.

### Study selection and data extraction

**Fig. 1:**
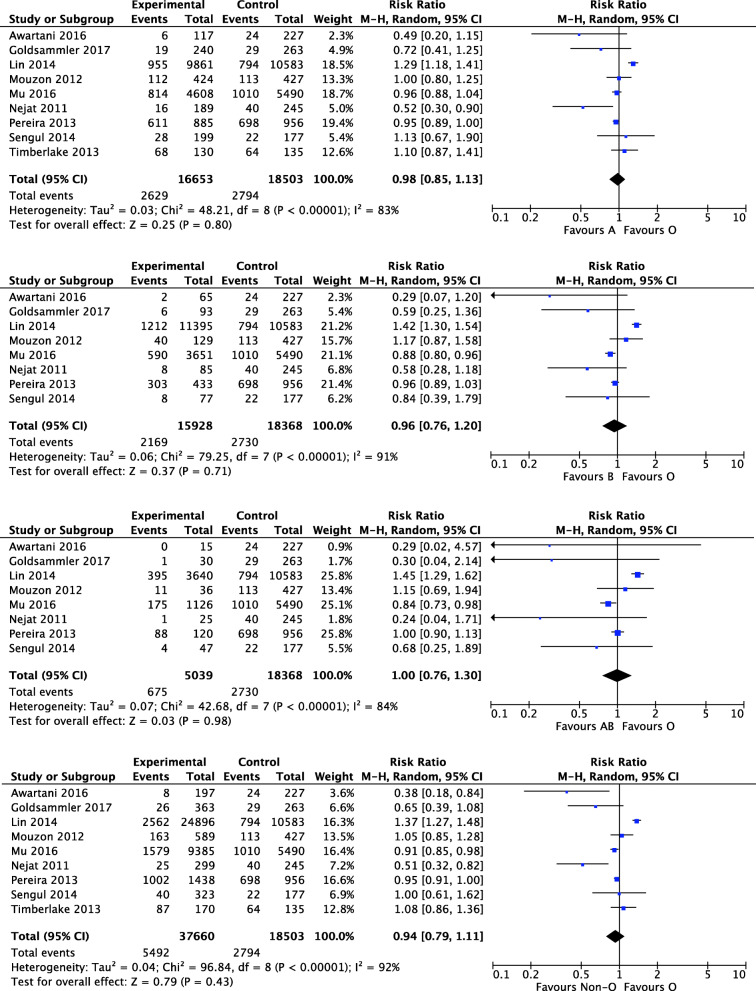
Forest plot showing the results of meta-analysis of studies assessing the association of ABO blood groups with DOR

After reviewing the retrieved titles and abstracts, irrelevant literatures were excluded. Then the full text of studies that may meet the criteria were reviewed and checked for eligibility. Articles that satisfying the above inclusion criteria were selected. Figure 1 presents the results of this search. The quality of the included studies was assessed via the Newcastle-Ottawa Quality Assessment Scale. Data was extracted by two authors independently using pre-defined criteria (Number of cases with or without observational outcomes were clearly listed in the article, and the women were all underdoing ART treatment). If any discrepancies are found, the opinion of the third author will be sought. Data extraction includes research features and results data.

### Statistical analysis

The present study used Review Manager version 5.3 for meta-analysis, and followed PRISMA guidelines whenever possible. Categorical variables were calculated using the Mantel-Haenszel statistical method and expressed as risk ratio (RR) values; Forest figures were used to graphically assess the heterogeneity of exposure effects, and I^2^ was used to statistically assess the heterogeneity of the entire study. A fixed or random-effect model was used to calculate the overall RR and its 95% confidence interval (CI). Because of the low power of the heterogeneity of the χ^2^ test when the sample size is small or the number of studies included is small, a *P* value of 0.1 instead of 0.05 was used to determine statistical significance.

## Results

### Studies selection and characteristics

**Fig. 2:**
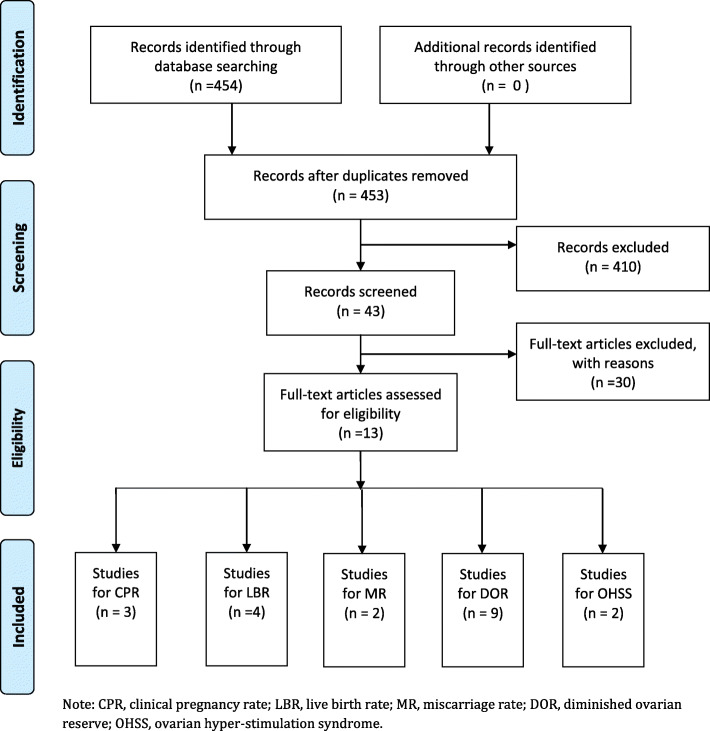
Flow chart showing study selection process. Note: CPR, clinical pregnancy rate; LBR, live birth rate; MR, miscarriage rate; DOR, diminished ovarian reserve; OHSS, ovarian hyper-stimulation syndrome

The literature retrieval strategy described as above obtained a total of 454 articles after restricting language and research objects. By reviewing the title and abstract, 411 articles were excluded because they were not relevant. Of the remaining 43 articles, 30 were excluded for different reasons: 20 reviews, 8 meta-analysis, and 2 with incomplete data. Finally, 13 articles were included in the present study (Fig. 2).

The 13 eligible studies were published from 2010 to 2018, including 8 retrospective studies, 2 prospective studies, 2 cross-section studies, and one study without study type reported. The sample size ranged from 305 to 35,479. Of 13 studies, 9, 2, 4, 3, 2 studies reported the association of ABO blood group with DOR, OHSS, LBR, CPR, and MR, respectively (Table 1).

**Table 1:** The characteristic of included studies

Author/year	Country	Type of study	Sample size	Inclusion criteria	Exclusion criteria	Groups	Outcomes
Nisio et al., 2018 [8]	Italy	Pros	497 patients	Age <40y; ≤3 embryo ET;	With anticoagulant treatment; without ET; PGS	A: 203 B: 63 AB: 18 O: 213	LBR; MR; CPR; positive pregnancy test;
Goldsammler et al., 2015 [7]	USA	Retro	626 patients	NR	NR	A: 117 B: 65 AB: 15 O: 227	No. of women with FSH> 10; LBR
Pereira et al., 2017 [1]	USA	Retro	2329 patients	Normal responders, bFSH≤12;AMH≥1 ng/mL; Age<40y; 1st IVF with SET	PCOS; Age ≥40y; with donor oocytes; PGS	A: 897 B: 397 AB: 120 O: 1915	LBR; Birth weight; Gestational age
Awartani et al., 2016 [16]	Saudi Arabia	Retro	424 patients with 566 IVF cycles	Age <40y;		A: 117 B: 65 AB: 15 O: 227	No. of women with FSH> 10; No. of oocytes retrieved; CPR
Mu et al., 2016 [10]	China	Retro	14,875 patients	NR	With missing data or >45y	A: 4608 B: 3651 AB: 1126 A antigen: 5734 B antigen: 4777 O: 5490	No. of women with FSH> 10
Lin et al., 2014 [11]	China	Retro	35,479 patients	Age ≤45y;	NR	A: 9861 B: 11395 AB: 3640 O: 10583	No. of women with FSH> 10
Sengul et al., 2014 [14]	Turkey	Pros	500	Age 18-45y;	NR	A: 424 B: 129 AB: 36 O: 427	No. of women with FSH≥10
Spitzer et al., 2014 [15]	Austria	Retro	1202 patients with 1889 IVF cycles	Only Caucasian	Lost to follow-up	A: 291+ 243 B: 86+ 64 AB: 37+ 31 O: 244+ 206	No. of COS/ MII; CPR; ongoing pregnancy rate
Pereira et al., 2013 [21]	USA	Retro	2394 cycles	DOR	NR	A: 885 B: 433 AB: 120 O: 956	No. of women with AMH< 1.5 ng/mL
Timberlake et al., 2013 [13]	North Carlina	Cross- sectional	305 patients	NR	NR	A: 189 B: 85 AB: 25 O: 24	No. of women with FSH> 10
Mouzon et al., 2012 [12]	France	Retro	1016 patients	NR	NR	A: 424 B: 129 AB: 36 O: 427	No. of women with AMH< 1.5 ng/mL
Nejat et al., 2011 [9]	USA	Cross- sectional	544 patients	Age ≤45y;	NR	A: 189 B: 85 AB: 25 O: 245	No. of women with FSH> 10;
Bellver et al., 2010 [20]	Spain	NR	842	At risk of early OHSS (> 20 oocytes) after IVF	NR	A: 376 B: 56 AB: 33 O: 377	No. of OHSS

Note: *NR* Not referred

### Meta-analysis

Our meta-analysis included 9 studies to assess the relationship between ABO blood group and DOR in infertile women undergoing ART treatment. The results showed the incidence of DOR in women with blood group O was no significantly different compared with those with blood group A (RR, 0.98; 95%CI 0.85, 1.13, *P*=0.80), blood group B (RR, 0.96; 95%CI 0.76, 1.20, *P*=0.71), blood group AB (RR, 1.00; 95%CI 0.76, 1.30, *P*=0.98), and non-O blood group (RR, 0.94; 95%CI 0.79, 1.11, *P*=0.43). The I^2^s, which were used to describe the heterogeneity of the included studies, were all > 75%. The results demonstrated highly heterogeneous between studies and the random effect model was used (Fig. 1).

**Fig. 3:**
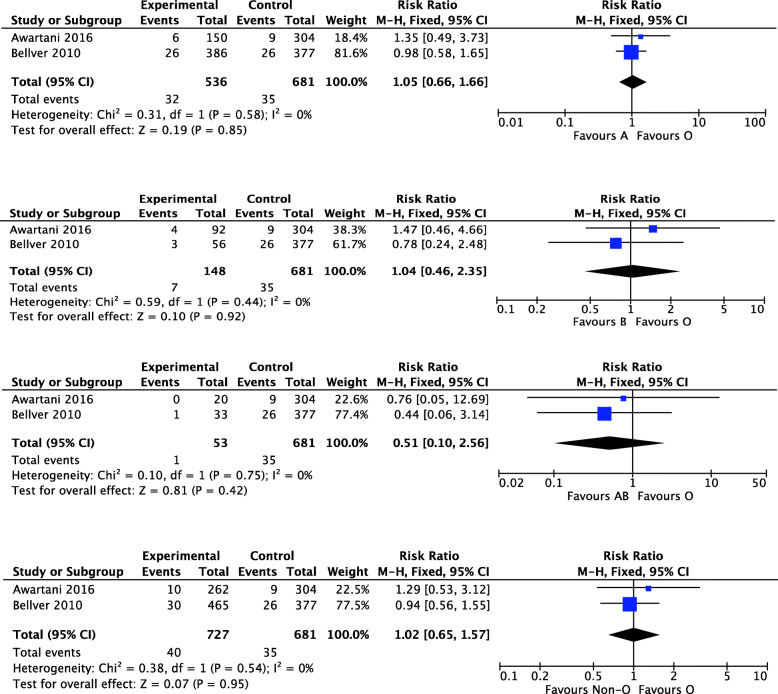
Forest plot showing the results of meta-analysis of studies assessing the association of ABO blood groups with OHSS

Similarly, two studies were included to exam the relationship between ABO blood group and OHSS. The result of the meta-analysis showed similar incidence of OHSS in women with blood group A/ B/ AB/ non-O and women with blood group O. The I^2^s were all 0%, indicating good heterogeneity. The fixed-effect models were used and the combined RRs were (RR, 1.05; 95%CI 0.66, 1.66, *P*=0.85), (RR, 1.04; 95%CI 0.46, 2.35, *P*=0.92), (RR, 0.51; 95%CI 0.10, 2.56, *P*=0.42), (RR, 1.02; 95%CI 0.65, 1.57, *P*=0.95), respectively (Fig. 3).

**Fig. 4:**
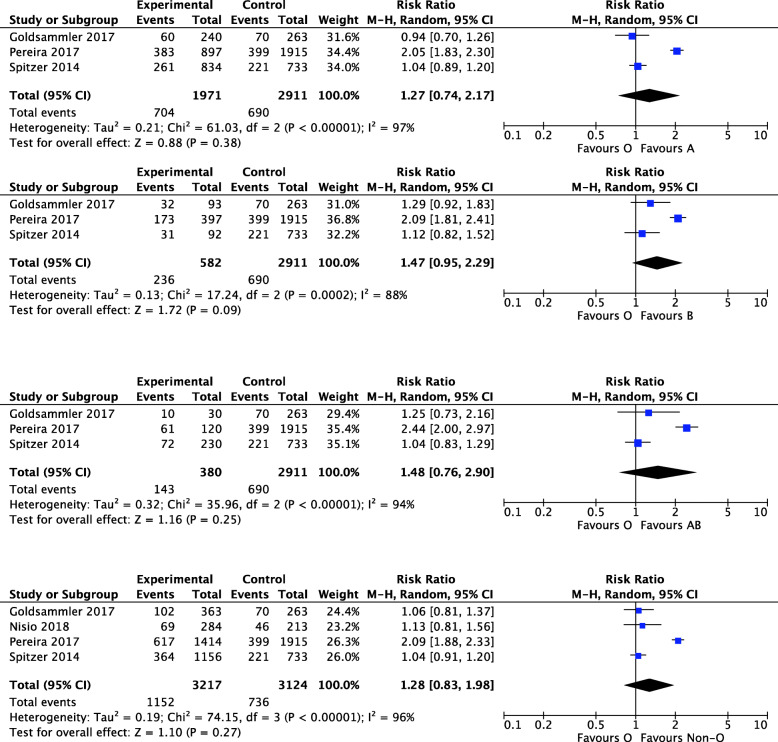
Forest plot showing the results of meta-analysis of studies assessing the association of ABO blood groups with LBR

With regard to the pregnancy outcomes, the LBR of women with blood group A/B/AB/non-O were not different from women with blood group O. There were significant homogeneity in the studies because I^2^s were all > 75%. The random effect models were used and the combined RRs were 1.27 (95%CI 0.74, 2.17, *P*=0.38), 1.47 (95%CI 0.95, 2.29, *P*=0.09), 1.48 (95%CI 0.76, 2.90, *P*=0.25), 1.28 (95%CI 0.83, 1.98, *P*=0.27), respectively (Fig. 4). Compared with women with blood group O, women with blood group A/ B/ AB/ non-O had similar CPR. The combined RRs were 1.12 (95%CI 0.90, 1.38, *P*=0.31), 1.08 (95%CI 0.89, 1.30, *P*=0.43), 1.05 (95%CI 0.90, 1.24, *P*=0.52), 1.05 (95%CI 0.96, 1.15, P=0.27), respectively (Sup Fig. 1). At last, two studies compared the MR between women with blood group A and blood group non-O. There was good homogeneity (I^2^=0%, *P*=0.33), and the combined RR was 0.94 (95%CI 0.76, 1.18, *P*=0.62) with fix effect model (Sup Fig. 2).

All of the Meta results were summarized in Table 2.

**Table 2:** summarized results of meta-analysis for women infertility

Outcome	Studies	Participants	Statistical Method	Effect Estimate	*P* value	Homogeneity(I^2^)	*P* value
DOR							
A vs. O	9	35,156	RR (M-H, Random, 95%CI)	0.98[0.85,1.13]	0.80	85%	< 0.00001
B vs. O	8	34,296	RR (M-H, Random, 95%CI)	0.96[0.76,1.20]	0.71	91%	< 0.00001
AB vs. O	8	23,407	RR (M-H, Random, 95%CI)	1.00[0.76,1.30]	0.98	84%	< 0.00001
Non-O vs. O	9	35,163	RR (M-H, Random, 95%CI)	0.94[0.79,1.11]	0.43	92%	< 0.00001
OHSS							
A vs. O	2	1217	RR (M-H, Fixed, 95%CI)	1.05[0.66,1.66]	0.85	0%	0.58
B vs. O	2	829	RR (M-H, Fixed, 95%CI)	1.04[0.46,2.35]	0.92	0%	0.44
AB vs. O	2	734	RR (M-H, Fixed, 95%CI)	0.51[0.10,2.56]	0.42	0%	0.75
Non-O vs. O	2	1408	RR (M-H, Fixed, 95%CI)	1.02[0.65,1.57]	0.95	0%	0.54
LBR							
A vs. O	3	4882	RR (M-H, Random, 95%CI)	1.27[0.74,2.17]	0.38	97%	< 0.00001
B vs. O	3	3493	RR (M-H, Random, 95%CI)	1.47[0.95,2.29]	0.09	88%	0.0002
AB vs. O	3	3291	RR (M-H, Random, 95%CI)	1.48[0.76,2.90]	0.25	94%	< 0.00001
Non-O vs. O	4	6341	RR (M-H, Random, 95%CI)	1.28[0.83,1.98]	0.27	96%	< 0.00001
CPR							
A vs. O	2	1965	RR (M-H, Random, 95%CI)	1.12[0.90,1.38]	0.31	61%	0.11
B vs. O	2	1194	RR (M-H, Fixed, 95%CI)	1.08[0.89,1.30]	0.43	0%	0.99
AB vs. O	2	1266	RR (M-H, Fixed, 95%CI)	1.05[0.90,1.24]	0.52	17%	0.27
Non-O vs. O	3	2886	RR (M-H, Fixed, 95%CI)	1.05[0.96,1.15]	0.27	8%	0.34
MR							
Non-O vs. O	2	2386	RR (M-H, Fixed, 95%CI)	0.94[0.76,1.18]	0.62	0%	0.33

Note: *DOR* Diminished ovarian reserve; *OHSS* Ovarian hyperstimulation syndrome; *CPR* Clinical pregnancy rate; *LBR* Live birth rate; *MR* Miscarriage rate; *RR* Risk ratio

The studies included in this meta-analysis scored medium to high basing on the Newcastle-Ottawa Scale, NOS (not shown). The meta-analysis funnel chart assessed the publication biases of included studies examining the association between ABO blood group and DOR, OHSS, pregnancy outcomes after ART. Due to their imperfect symmetrical shapes, these studies showed possible publication bias. (Sup Fig. 1.1–5.1).

## Discussion

This study was the first meta-analysis of the association between ABO blood group and DOR, OHSS, and IVF pregnancy outcomes. The results suggested that there is no significant difference in the incidence of DOR, OHSS, LBR, CPR, and MR between blood group A/B/AB/non-O and blood group O.

The ABO blood group gene is located on chromosome 9q34 and has three main allele forms: the A allele, the B allele, and the O allele [22]. The A/B allele encodes a glycosyltransferase (A/B transferase), which catalyzes the transfer of nucleotide sugars to H antigens, thereby forming A/B blood group antigens [23]. The O allele has a single base deletion in the coding region near the N-terminus of the protein, the product is a protein with no enzyme activity, and the H antigen remains unchanged on the red blood cell [24].

Studies have found that the blood group was related to ovarian reserve. The possible explanations were speculated as following: (1) FSH and LH receptors are glycosylated proteins, which are essential for follicular development and maturation. The biological activities of FSH and LH are likely to be altered by the sugar transferase encoded by the O allele [23]. Besides, glycotransferase could affect the half-life and biological activity of LH [25]. (2) Some ovarian function related genes located near the ABO locus, such as nuclear receptor 5A1 (NR5A1) and transforming growth factor beta-receptor (TGFBR1) genes [26]. Therefore, these genes and ABO-group genes may recombine and be inherited together with ABO. (3) Haplotype DNA mutations can change the stability of folded proteins, thereby making certain allele combinations more commonly inherited together [27]. (4) In the last, other genetic factors such as FSH receptor polymorphism [28] and the fragile X mental retardation 1 gene (FMR1) rank carrier status [29] are associated with elevated FSH levels. Recent genome-wide association studies have identified about 20 loci related to the women menopause [30].

However, ABO antigens reflect ancient polymorphisms shared by many primates [31]. If there is a mutation or polymorphism of an unknown gene that causes DOR and is related to the ABO locus, it is likely to be younger than the ABO locus. Therefore, if there is such a mutation, although it may be scattered in some subgroups, it will not be scattered on all O antigen carriers. Therefore, any correlation between ABO blood group and ovarian reserve can be ignored.

Correspondingly, a good ovarian reserve is likely to have a high response to hyper- ovulation, even develop to OHSS. In addition, previous studies have suggested that women with blood group A are more likely to have ovulation disorders, and ovulation disorders are considered to be a high-risk factor for OHSS [18]. Women of blood group A are more likely to develop early-onset OHSS, and the incidence of early-onset OHSS of blood group O is lower. This finding may be due to the 25% lower plasma concentrations of Von Willebrand factor and coagulation factor VIII in individuals with blood group O compared with the blood group A [32]. However, the increase of coagulation factors in plasma is one of the high-risk factors for the OHSS, but it is not an absolute cause. Endothelial dysfunction was another important factor for OHSS. Endothelial dysfunction increases the permeability of capillaries, resulting in fluid loss into the third space, and subsequent changes in metabolism and hematology [33]. For people with non-O blood group, two-thirds of the overall change in VWF concentration seems to be genetically determined, with higher concentrations of VWF and factor VIII [34]. According to reports, in many different clinical situations such as HELLP [35] and OHSS, VWF levels are elevated and endothelial cell dysfunction is present.

Regarding the relationship between the ABO blood group and pregnancy outcomes, previous studies have found that a certain blood group is less likely to achieve a successful pregnancy. As mentioned above, the ABO blood group is the main determinant of plasma von Willebrand factor and factor VIII levels, and hemoglobin and factor VIII levels increase in individuals with non-O blood groups [36, 37]. Recently, one study proposed that the ADAMTS13-von Willebrand factor pathway play a key role in normal pregnancy and pathogenesis of preeclampsia [38]. Multiple studies have also shown that certain ABO blood group gene polymorphisms are associated with increased levels of some immune and inflammatory mediators [39, 40], which are associated with early embryo implantation and subsequent placental implantation [41, 42]. Therefore, it is reasonable to believe that the immune or inflammatory environment associated with certain ABO blood groups may affect the embryo implantation and subsequent embryo growth during IVF.

With regard to the relationship between ABO blood group and DOR, OHSS, pregnancy outcomes, the conclusions were inconsistent, and even opposite. The contradiction may be due to the racial differences between the study populations. Even the study populations were from the same country; there may be different results, which may be related to the difference in the blood group composition ratio of the study population.

There were several limitations in our present study. The biggest one was that 11 out of the 13 included studies were retrospective studies. When evaluating some outcomes such as MR and OHSS, relatively few studies were included. Secondly, there was highly variable study characteristic: the diagnosis standard for DOR, population racial (Ethnicity and race also affect the distribution of different blood groups [43]). Thirdly, some studies did not adjust for some possible confounding factors, such as smoking history, body mass index, and history of ovarian surgery.

## In conclusion

The present study indicated that ABO blood groups might not be associated with the incidence of DOR, OHSS, LBR, CPR, and MR after ART treatment. Infertility and ART outcomes are influenced by multiple factors. Blood groups should not be taken into account excessively during diagnosis and treatment of infertile women. Further well-designed clinical studies are needed to confirm the association between ABO blood group and women infertility.

## Supplementary Information

**Additional file 1: Fig. S1** Forest plot showing the results of meta-analysis of studies assessing the association of ABO blood groups with CPR. **Fig. S2** Forest plot showing the results of meta-analysis of studies assessing the association of ABO blood groups with MR.

**Additional file 2: Fig. S1.1** Funnel plot of analysis for the association of blood group A/O and DOR, showing the results of Eggers to assess publication bias. **Fig. S1.2** Funnel plot of analysis for the association of blood group B/O and DOR, showing the results of Eggers to assess publication bias. **Fig. S1.3** Funnel plot of analysis for the association of blood group AB/O and DOR, showing the results of Eggers to assess publication bias. **Fig. S1.4** Funnel plot of analysis for the association of blood group non-O/O and DOR, showing the results of Eggers to assess publication bias.

**Additional file 3: Fig. S2.1** Funnel plot of analysis for the association of blood group A/O and OHSS, showing the results of Eggers to assess publication bias. **Fig. S2.2** Funnel plot of analysis for the association of blood group B/O and OHSS, showing the results of Eggers to assess publication bias. **Fig. S2.3** Funnel plot of analysis for the association of blood group AB/O and OHSS, showing the results of Eggers to assess publication bias. **Fig. S2.4** Funnel plot of analysis for the association of blood group non-O/O and OHSS, showing the results of Eggers to assess publication bias.

**Additional file 4: Fig. S3.1** Funnel plot of analysis for the association of blood group A/O and LBR, showing the results of Eggers to assess publication bias. **Fig. S3.2** Funnel plot of analysis for the association of blood group B/O and LBR, showing the results of Eggers to assess publication bias. **Fig. S3.3** Funnel plot of analysis for the association of blood group AB/O and LBR, showing the results of Eggers to assess publication bias. **Fig. S3.4** Funnel plot of analysis for the association of blood group non-O/O and LBR, showing the results of Eggers to assess publication bias.

**Additional file 5: Fig. S4.1** Funnel plot of analysis for the association of blood group A/O and CPR, showing the results of Eggers to assess publication bias. **Fig. S4.2** Funnel plot of analysis for the association of blood group B/O and CPR, showing the results of Eggers to assess publication bias. **Fig. S4.3** Funnel plot of analysis for the association of blood group AB/O and CPR, showing the results of Eggers to assess publication bias. **Fig. S4.4** Funnel plot of analysis for the association of blood group non-O/O and CPR, showing the results of Eggers to assess publication bias.

**Additional file 6: Fig. S5.1** Funnel plot of analysis for the association of blood group non-O/O and MR, showing the results of Eggers to assess publication bias.

## Data Availability

All data is available in this paper.

## Abbreviations

ART: Assisted reproductive technology
DOR: Diminished ovarian reserve
OHSS: Ovarian hyperstimulation syndrome
RR: Risk ratio
CPR: Clinical pregnancy rate
LBR: Live birth rate
MR: Miscarriage rate
CI: Confidence interval
