# Supplementary material for: Association of ABO blood groups with ovarian reserve, and outcomes after assisted reproductive technology: systematic review and meta-analyses
Source: Reprod Biol Endocrinol. 2021 Feb 6;19:20. doi: 10.1186/s12958-020-00685-x (PMC7866457; doi:10.1186/s12958-020-00685-x)
Supplement: Supplementary file 1 — Additional file 1: Fig. S1 Forest plot showing the results of meta-analysis of studies assessing the association of ABO blood groups with CPR. Fig. S2 Forest plot showing the results of meta-analysis of studies assessing the association of ABO blood groups with MR. [file 12958_2020_685_MOESM1_ESM.pptx]

## Slide 1
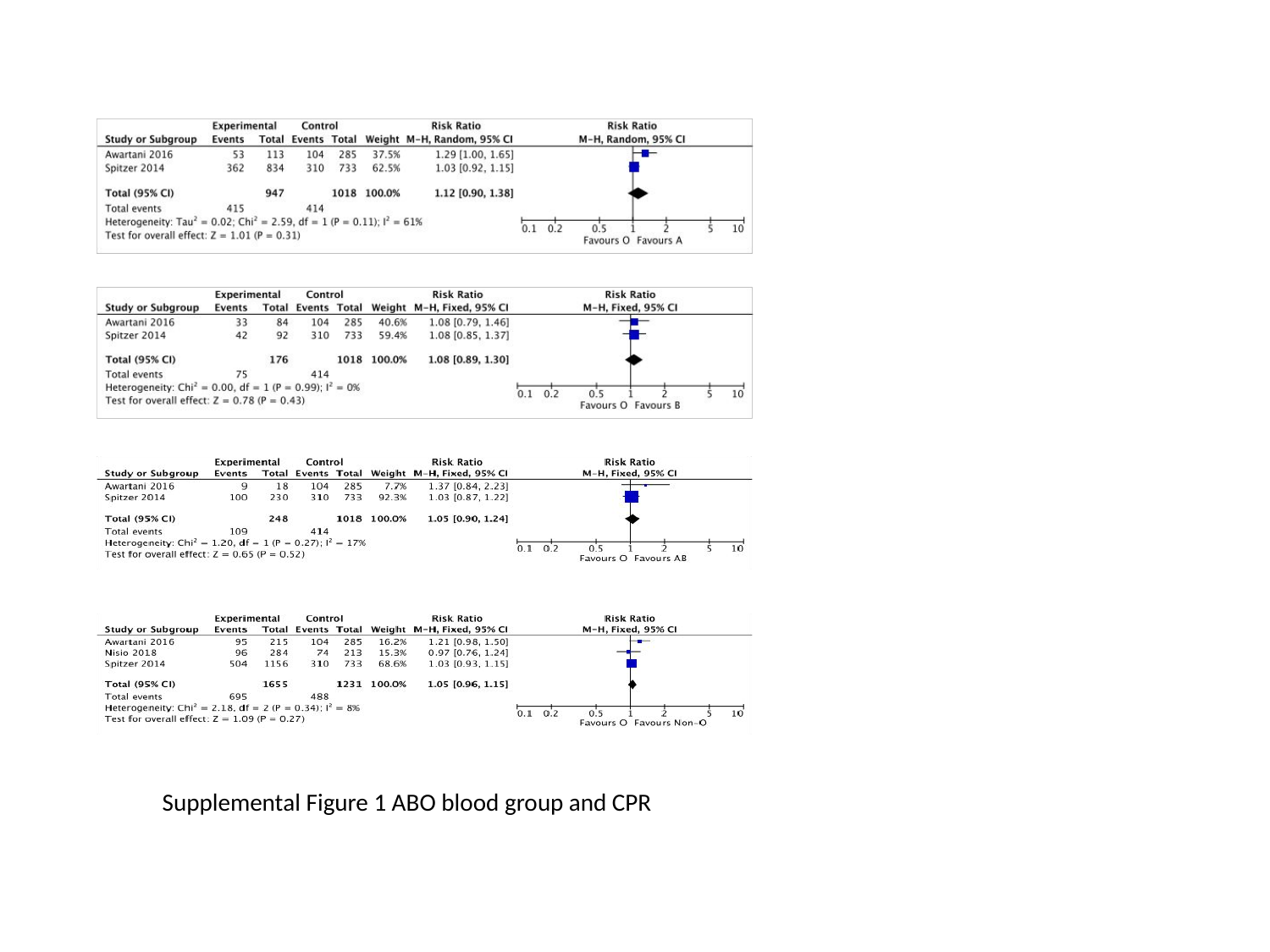

Supplemental Figure 1 ABO blood group and CPR

## Slide 2
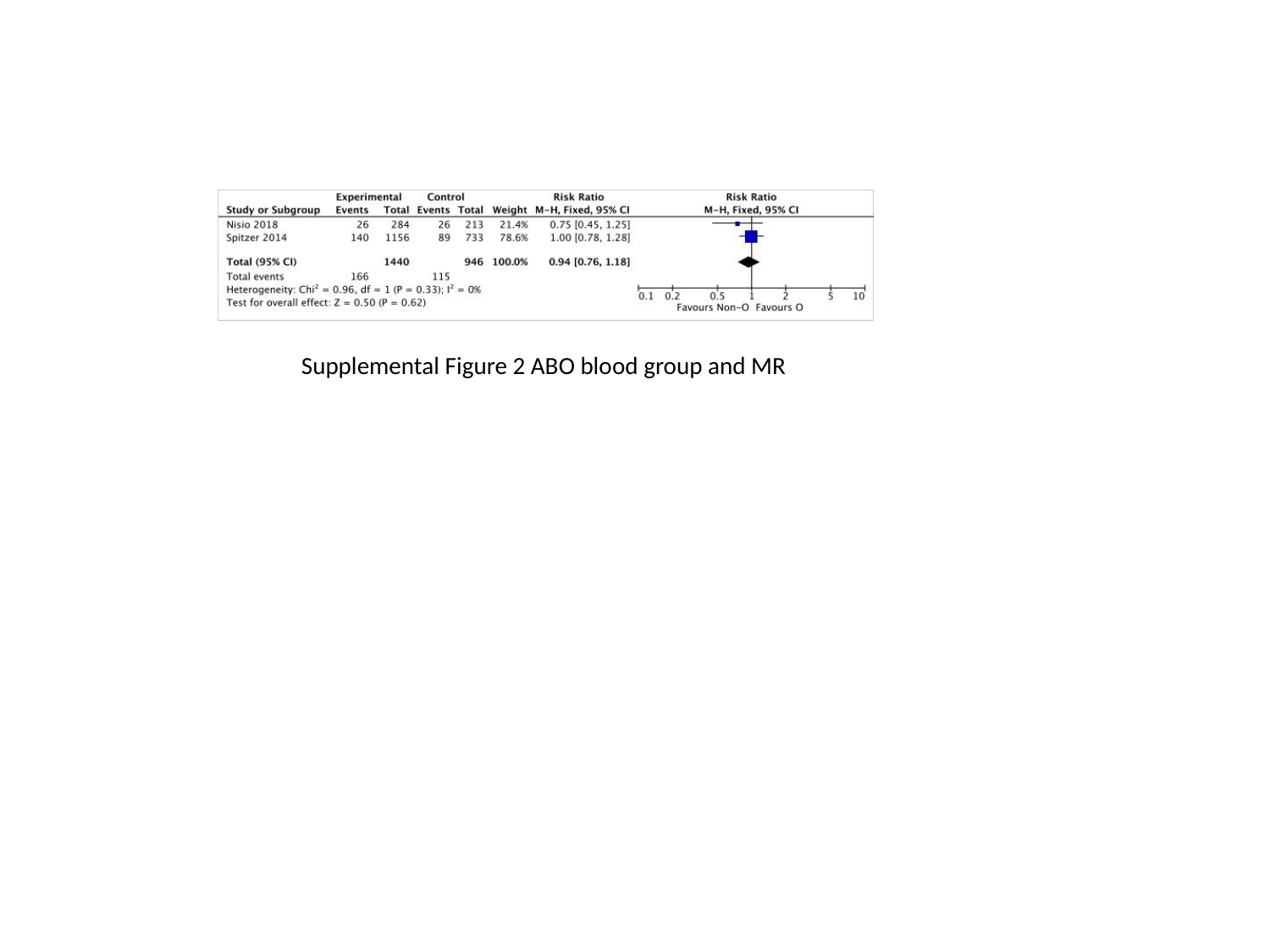

Supplemental Figure 2 ABO blood group and MR

## Slide 3
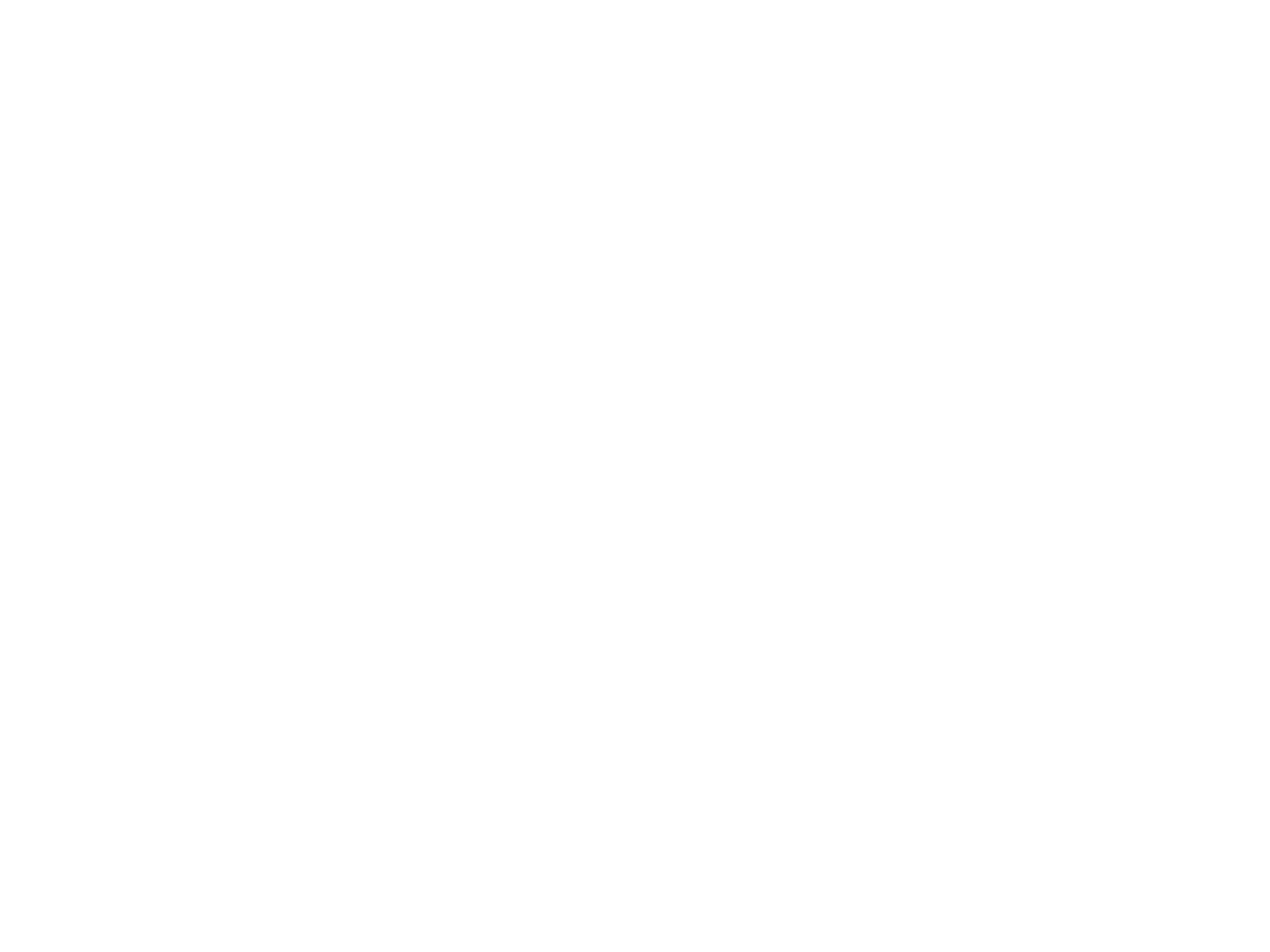

#

## Slide 4
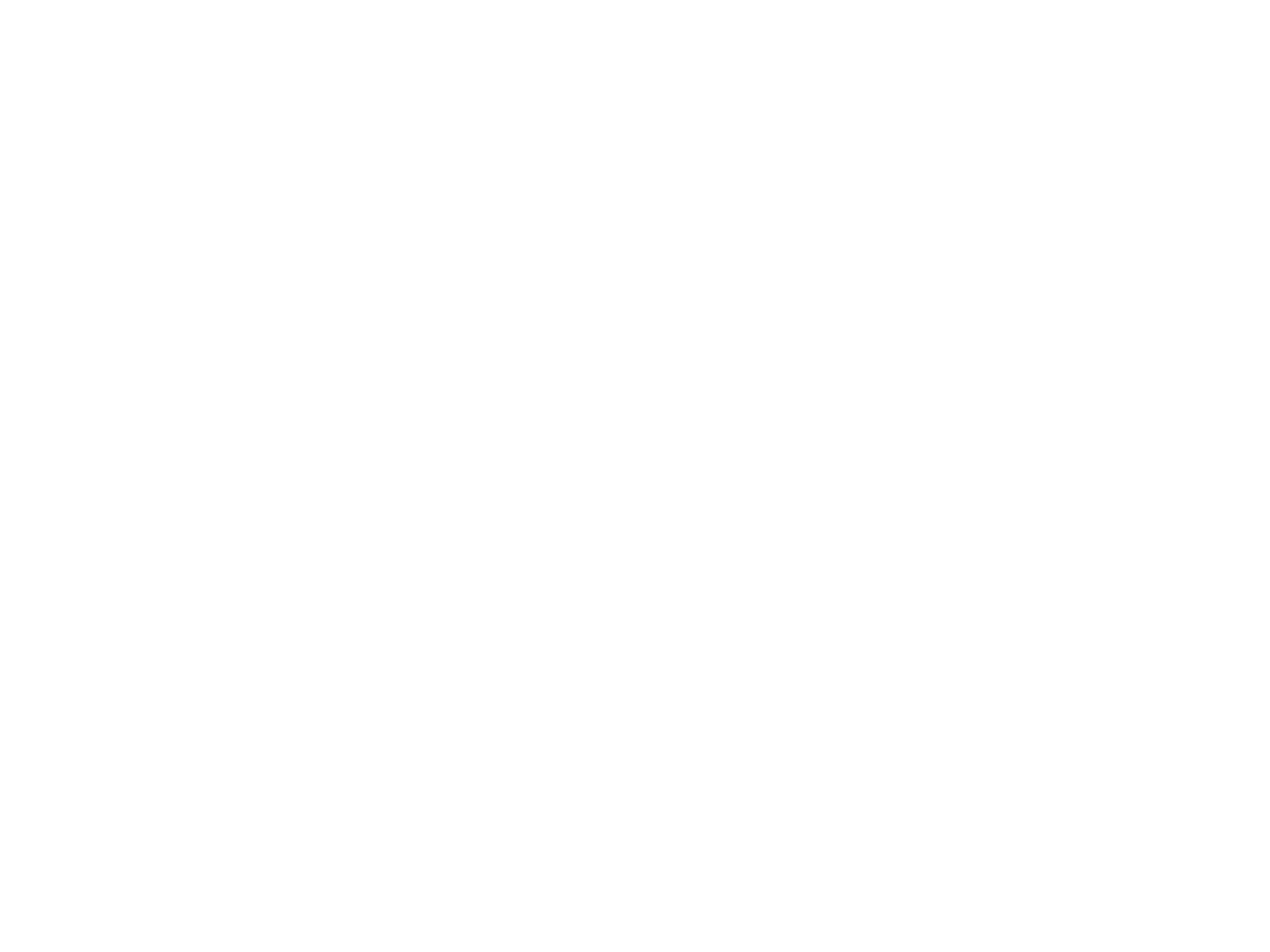

#
