# Supplementary figures and images for: Association of ABO blood groups with ovarian reserve, and outcomes after assisted reproductive technology: systematic review and meta-analyses
Source: Reprod Biol Endocrinol. 2021 Feb 6;19:20. doi: 10.1186/s12958-020-00685-x (PMC7866457; doi:10.1186/s12958-020-00685-x)

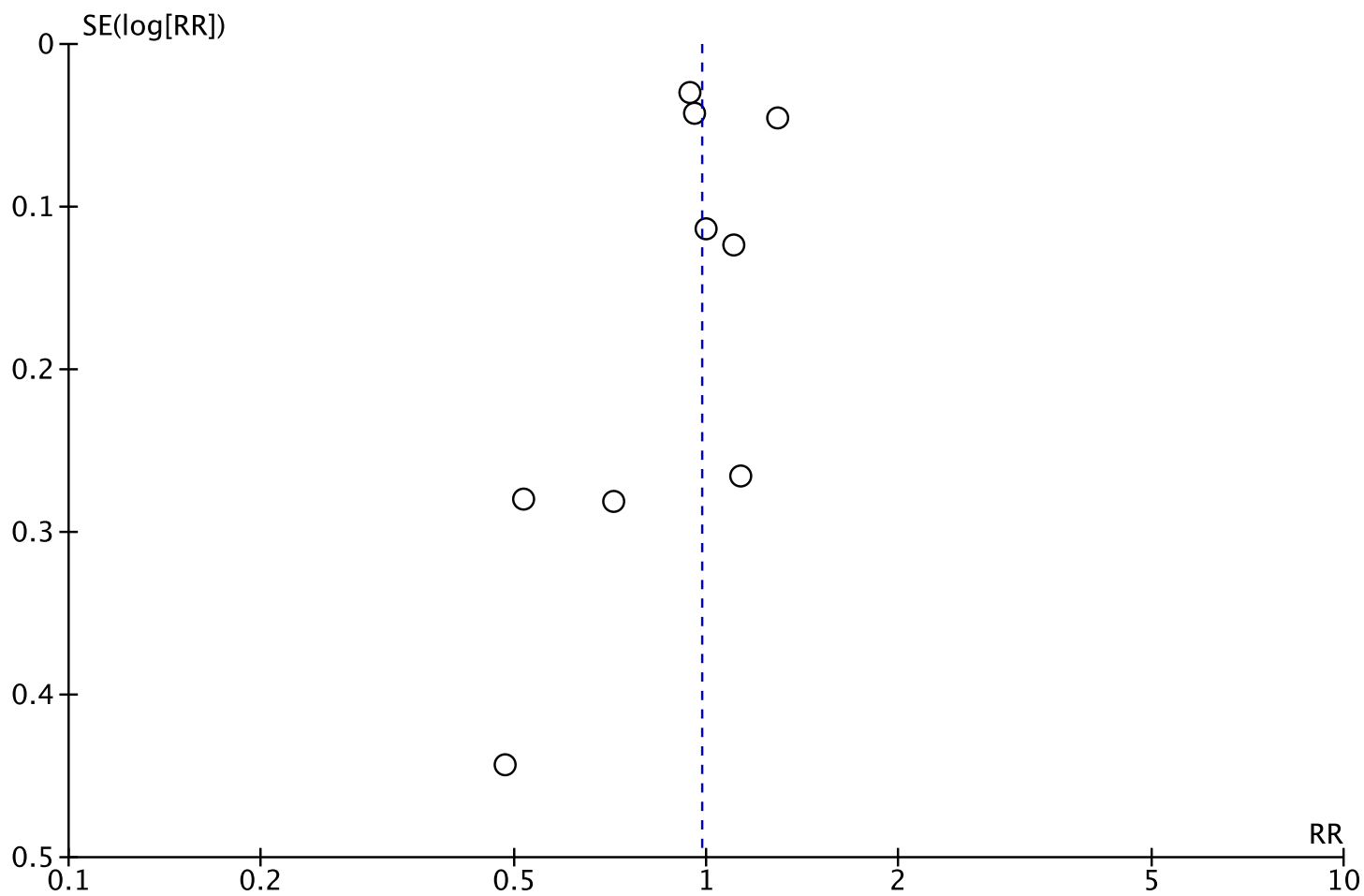

Supplement: Supplementary file 2 — Additional file 2: Fig. S1.1 Funnel plot of analysis for the association of blood group A/O and DOR, showing the results of Eggers to assess publication bias. Fig. S1.2 Funnel plot of analysis for the association of blood group B/O and DOR, showing the results of Eggers to assess publication bias. Fig. S1.3 Funnel plot of analysis for the association of blood group AB/O and DOR, showing the results of Eggers to assess publication bias. Fig. S1.4 Funnel plot of analysis for the association of blood group non-O/O and DOR, showing the results of Eggers to assess publication bias. [file 12958_2020_685_MOESM2_ESM.zip › 1.1 DOR A vs O Funnel plot.pdf]

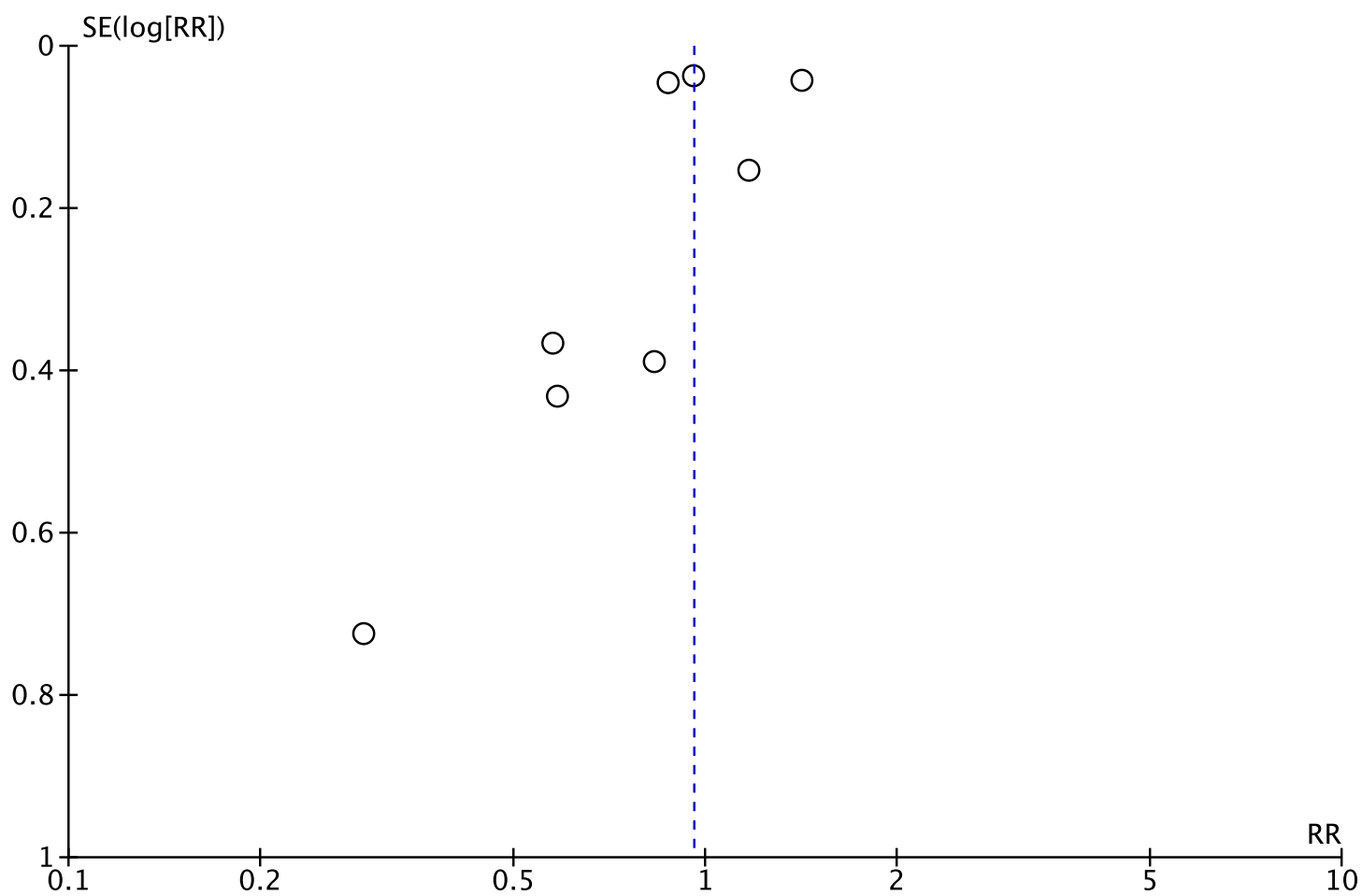

Supplement: Supplementary file 2 — Additional file 2: Fig. S1.1 Funnel plot of analysis for the association of blood group A/O and DOR, showing the results of Eggers to assess publication bias. Fig. S1.2 Funnel plot of analysis for the association of blood group B/O and DOR, showing the results of Eggers to assess publication bias. Fig. S1.3 Funnel plot of analysis for the association of blood group AB/O and DOR, showing the results of Eggers to assess publication bias. Fig. S1.4 Funnel plot of analysis for the association of blood group non-O/O and DOR, showing the results of Eggers to assess publication bias. [file 12958_2020_685_MOESM2_ESM.zip › 1.2 DOR B vs. O Funnel plot.pdf]

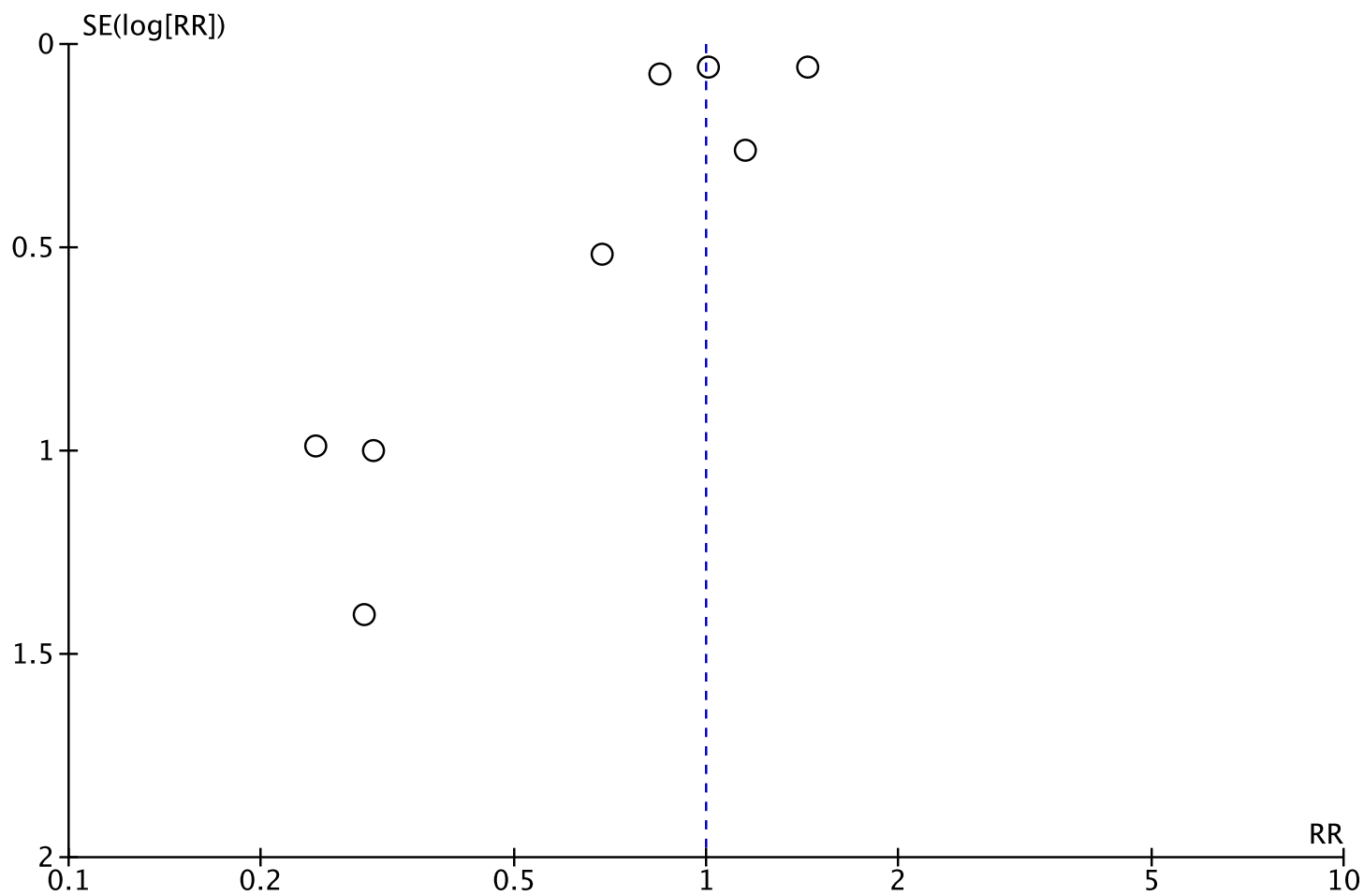

Supplement: Supplementary file 2 — Additional file 2: Fig. S1.1 Funnel plot of analysis for the association of blood group A/O and DOR, showing the results of Eggers to assess publication bias. Fig. S1.2 Funnel plot of analysis for the association of blood group B/O and DOR, showing the results of Eggers to assess publication bias. Fig. S1.3 Funnel plot of analysis for the association of blood group AB/O and DOR, showing the results of Eggers to assess publication bias. Fig. S1.4 Funnel plot of analysis for the association of blood group non-O/O and DOR, showing the results of Eggers to assess publication bias. [file 12958_2020_685_MOESM2_ESM.zip › 1.3 DOR AB vs. O Funnel plot.pdf]

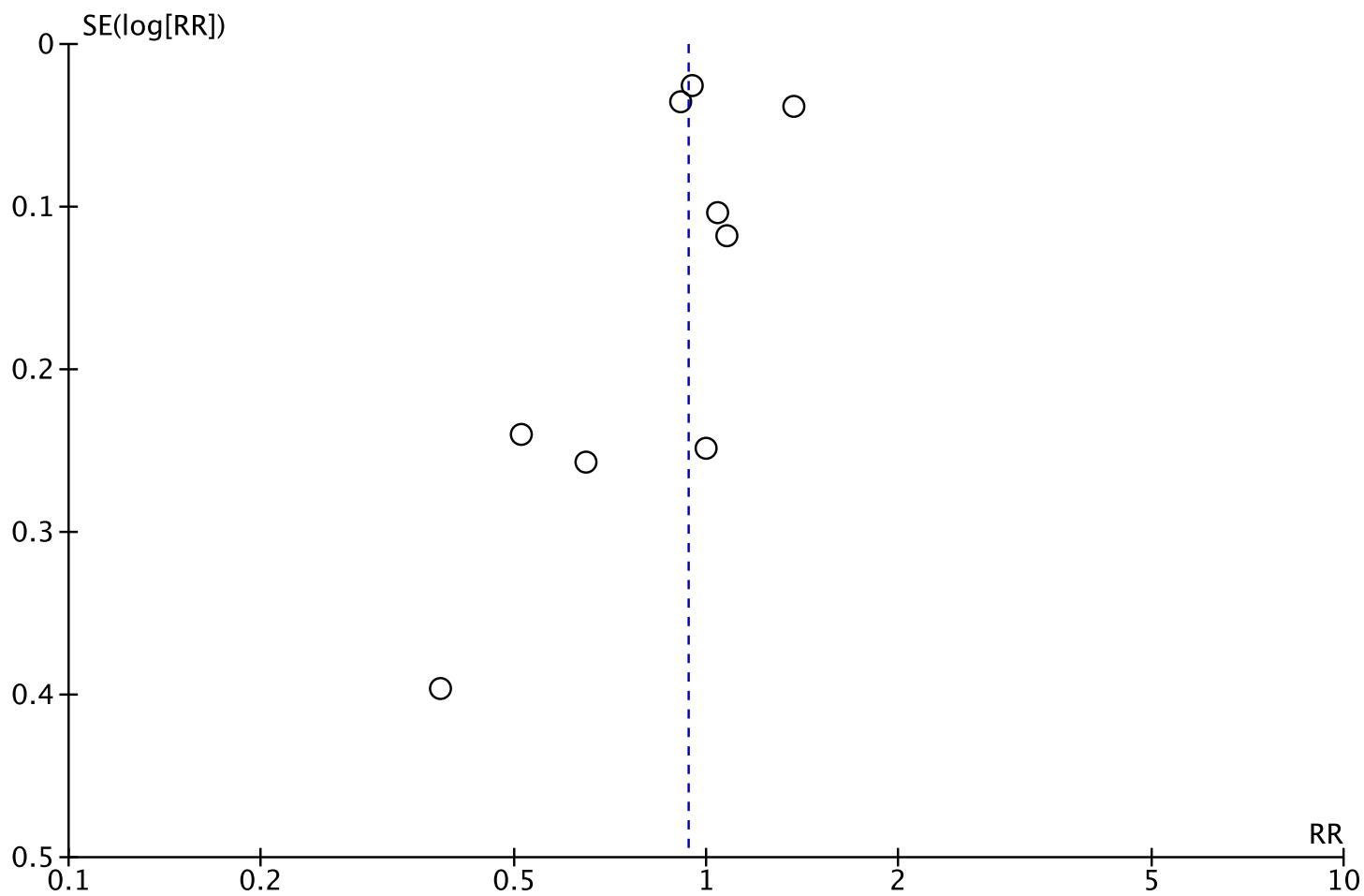

Supplement: Supplementary file 2 — Additional file 2: Fig. S1.1 Funnel plot of analysis for the association of blood group A/O and DOR, showing the results of Eggers to assess publication bias. Fig. S1.2 Funnel plot of analysis for the association of blood group B/O and DOR, showing the results of Eggers to assess publication bias. Fig. S1.3 Funnel plot of analysis for the association of blood group AB/O and DOR, showing the results of Eggers to assess publication bias. Fig. S1.4 Funnel plot of analysis for the association of blood group non-O/O and DOR, showing the results of Eggers to assess publication bias. [file 12958_2020_685_MOESM2_ESM.zip › 1.4 DOR Non-O vs. O Funnel plot.pdf]

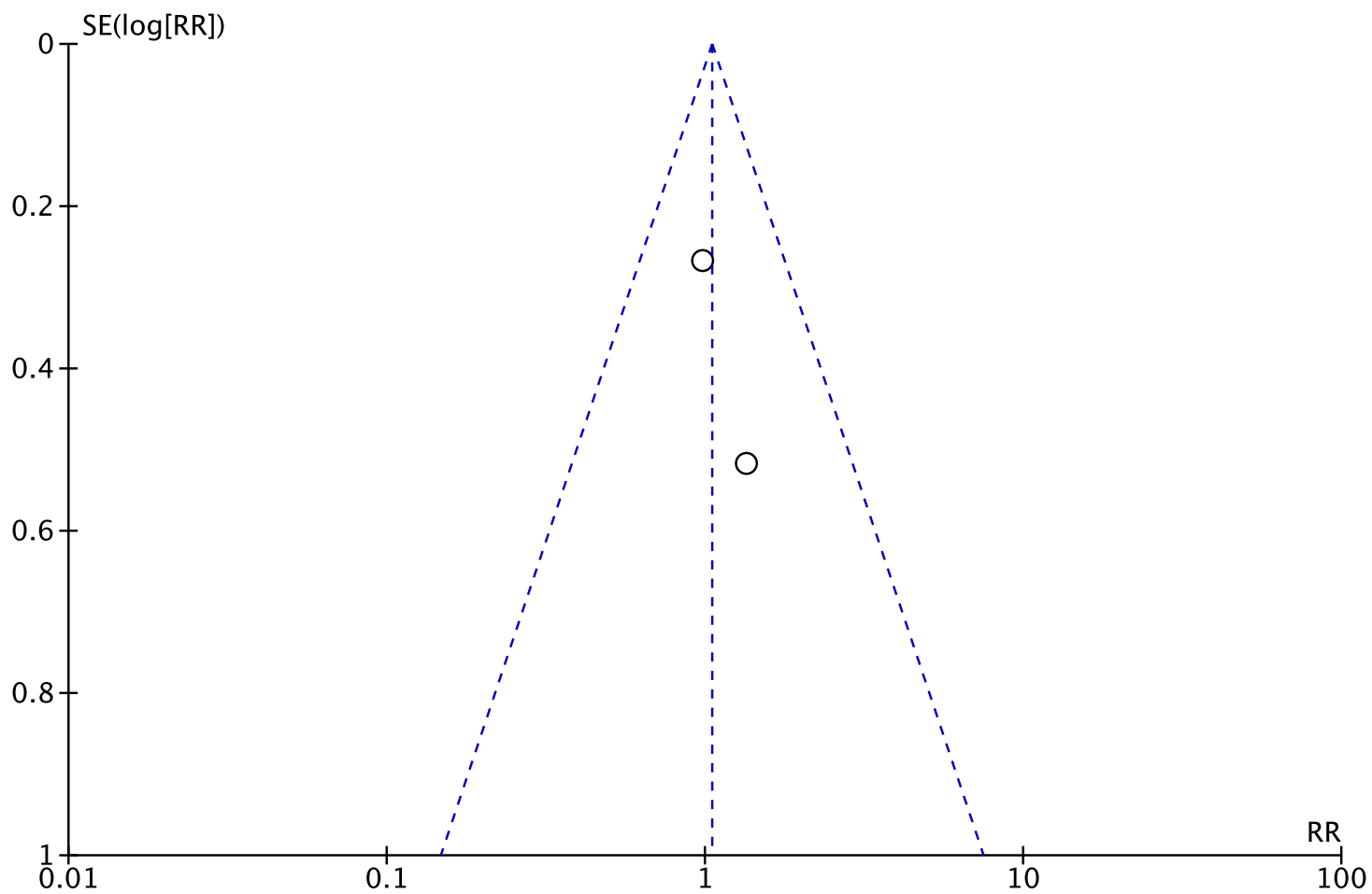

Supplement: Supplementary file 3 — Additional file 3: Fig. S2.1 Funnel plot of analysis for the association of blood group A/O and OHSS, showing the results of Eggers to assess publication bias. Fig. S2.2 Funnel plot of analysis for the association of blood group B/O and OHSS, showing the results of Eggers to assess publication bias. Fig. S2.3 Funnel plot of analysis for the association of blood group AB/O and OHSS, showing the results of Eggers to assess publication bias. Fig. S2.4 Funnel plot of analysis for the association of blood group non-O/O and OHSS, showing the results of Eggers to assess publication bias. [file 12958_2020_685_MOESM3_ESM.zip › 0715 2.1 OHSS A-O Funnel plot.pdf]

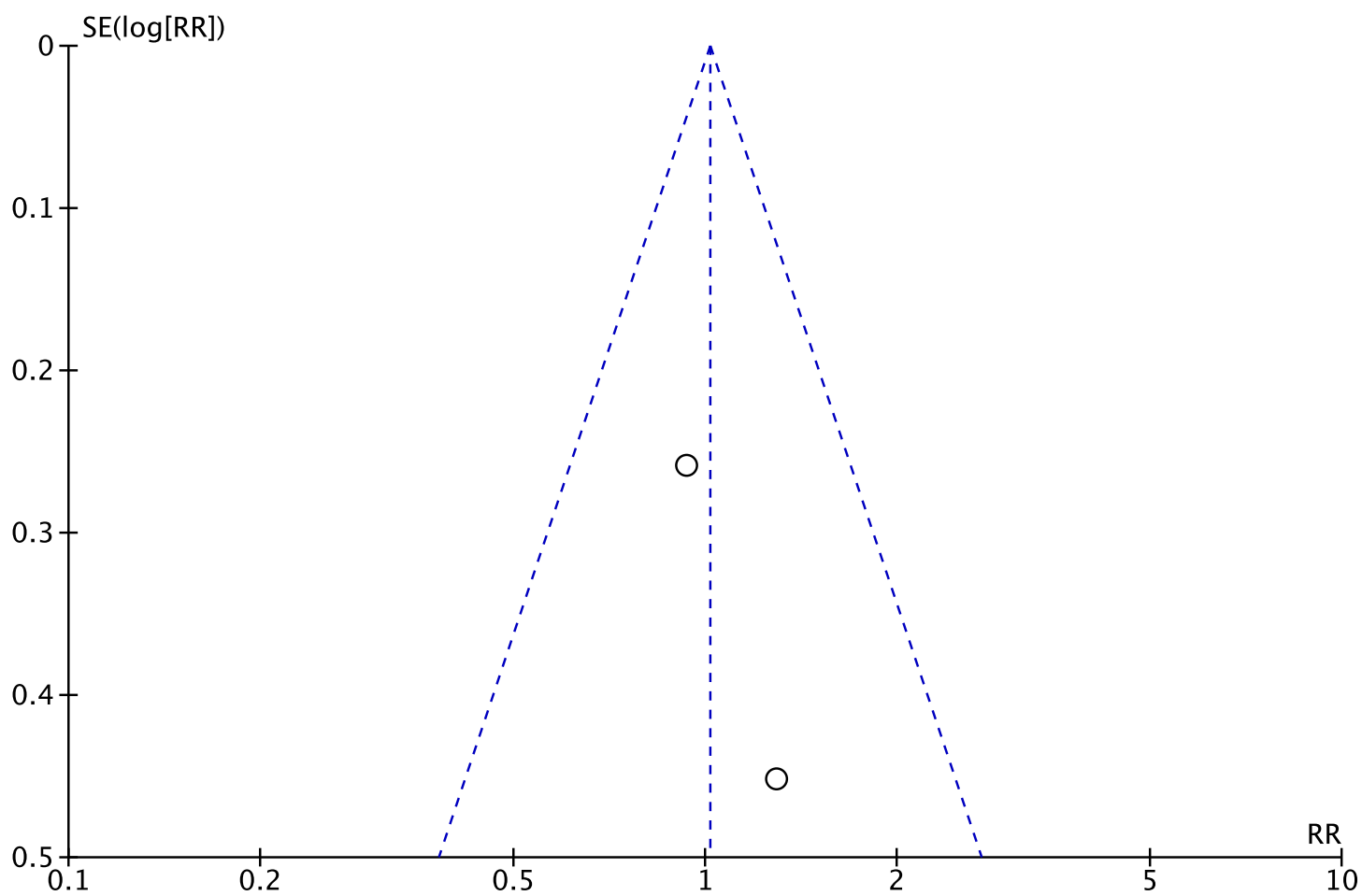

Supplement: Supplementary file 3 — Additional file 3: Fig. S2.1 Funnel plot of analysis for the association of blood group A/O and OHSS, showing the results of Eggers to assess publication bias. Fig. S2.2 Funnel plot of analysis for the association of blood group B/O and OHSS, showing the results of Eggers to assess publication bias. Fig. S2.3 Funnel plot of analysis for the association of blood group AB/O and OHSS, showing the results of Eggers to assess publication bias. Fig. S2.4 Funnel plot of analysis for the association of blood group non-O/O and OHSS, showing the results of Eggers to assess publication bias. [file 12958_2020_685_MOESM3_ESM.zip › 0715 2.4 OHSS NON-O-O Funnel plot.pdf]

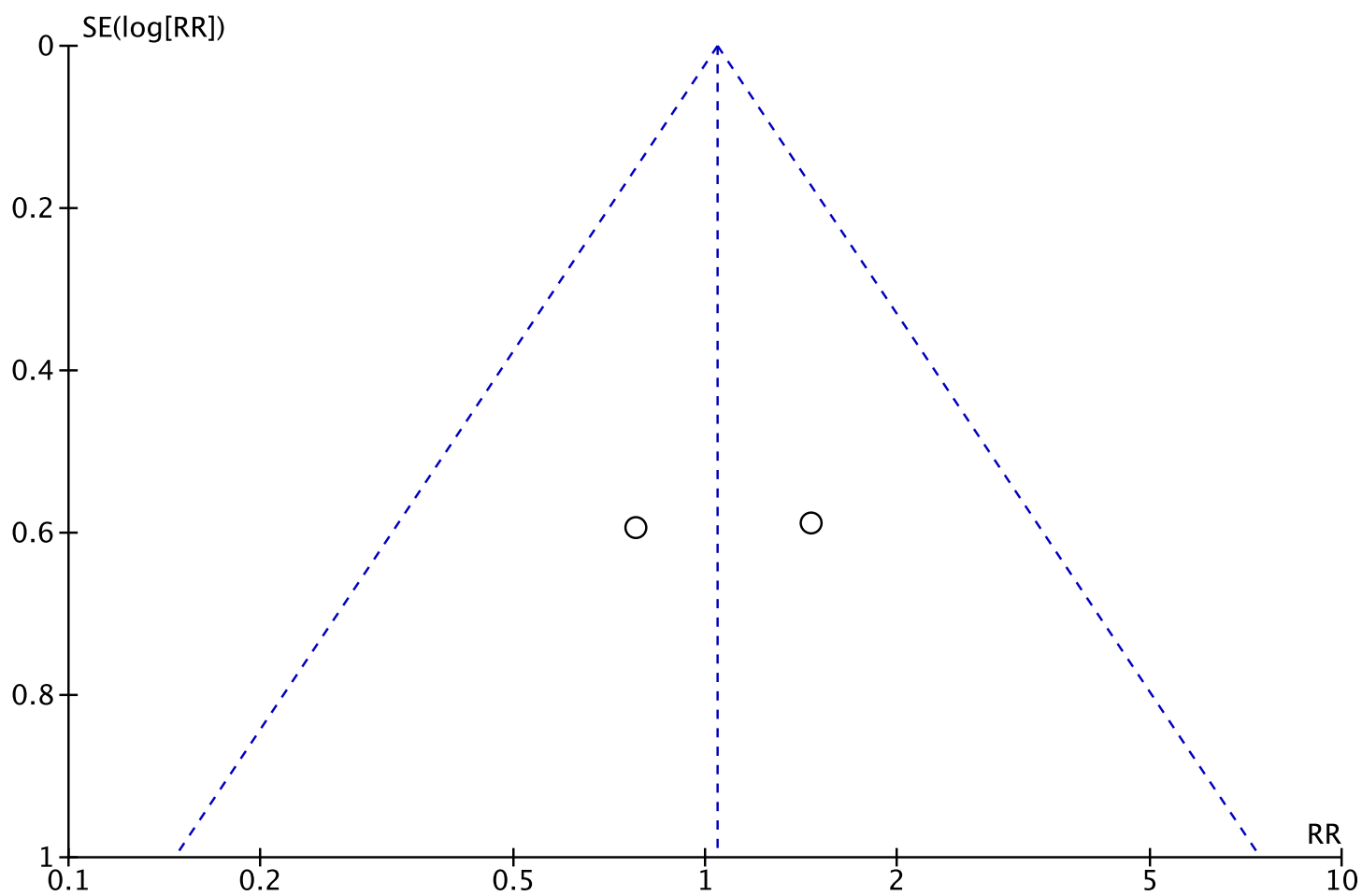

Supplement: Supplementary file 3 — Additional file 3: Fig. S2.1 Funnel plot of analysis for the association of blood group A/O and OHSS, showing the results of Eggers to assess publication bias. Fig. S2.2 Funnel plot of analysis for the association of blood group B/O and OHSS, showing the results of Eggers to assess publication bias. Fig. S2.3 Funnel plot of analysis for the association of blood group AB/O and OHSS, showing the results of Eggers to assess publication bias. Fig. S2.4 Funnel plot of analysis for the association of blood group non-O/O and OHSS, showing the results of Eggers to assess publication bias. [file 12958_2020_685_MOESM3_ESM.zip › 0715 OHSS 2.2 B-O Funnel plot.pdf]

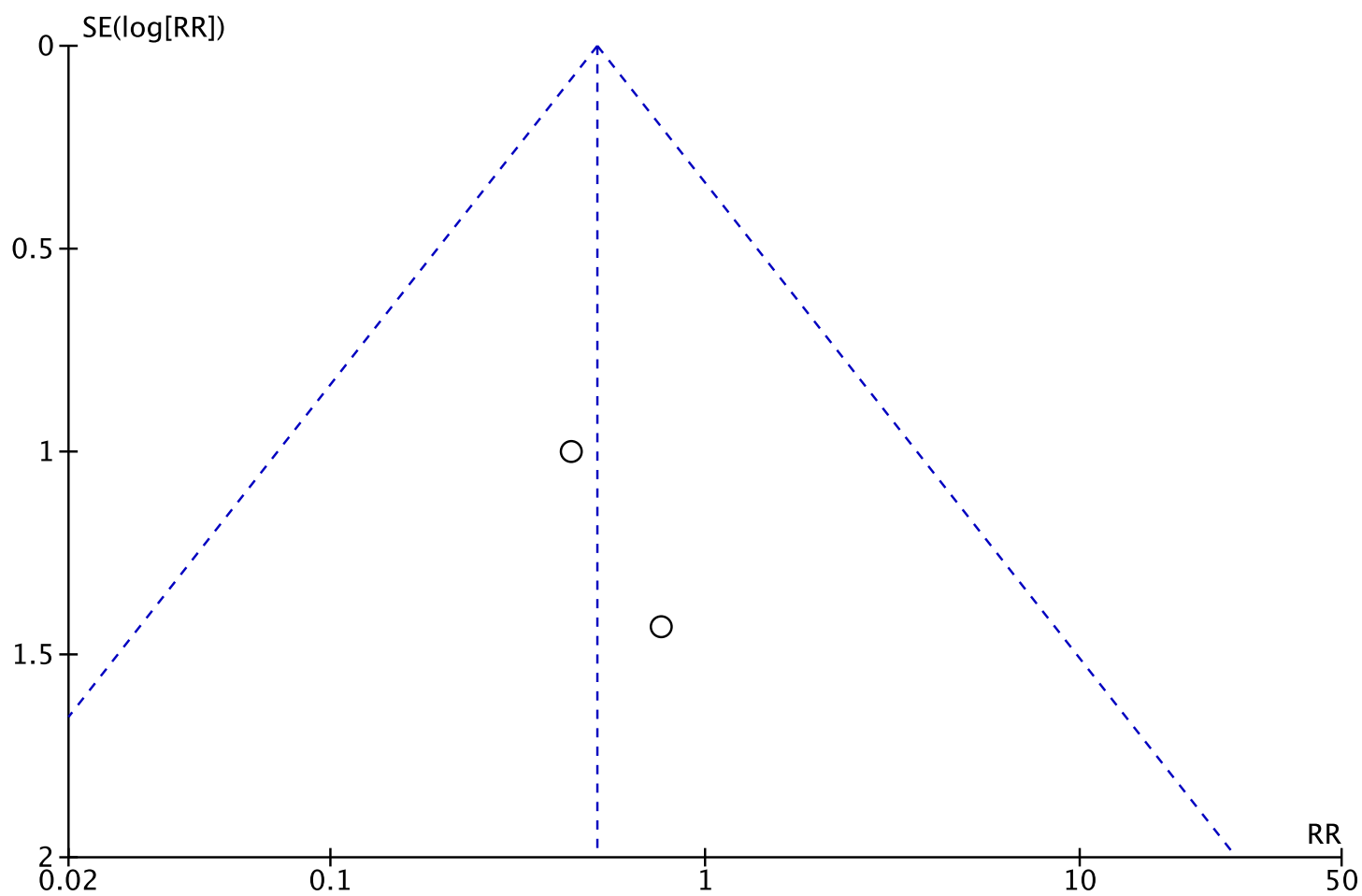

Supplement: Supplementary file 3 — Additional file 3: Fig. S2.1 Funnel plot of analysis for the association of blood group A/O and OHSS, showing the results of Eggers to assess publication bias. Fig. S2.2 Funnel plot of analysis for the association of blood group B/O and OHSS, showing the results of Eggers to assess publication bias. Fig. S2.3 Funnel plot of analysis for the association of blood group AB/O and OHSS, showing the results of Eggers to assess publication bias. Fig. S2.4 Funnel plot of analysis for the association of blood group non-O/O and OHSS, showing the results of Eggers to assess publication bias. [file 12958_2020_685_MOESM3_ESM.zip › 0715 OHSS 2.3 AB-O Funnel plot.pdf]

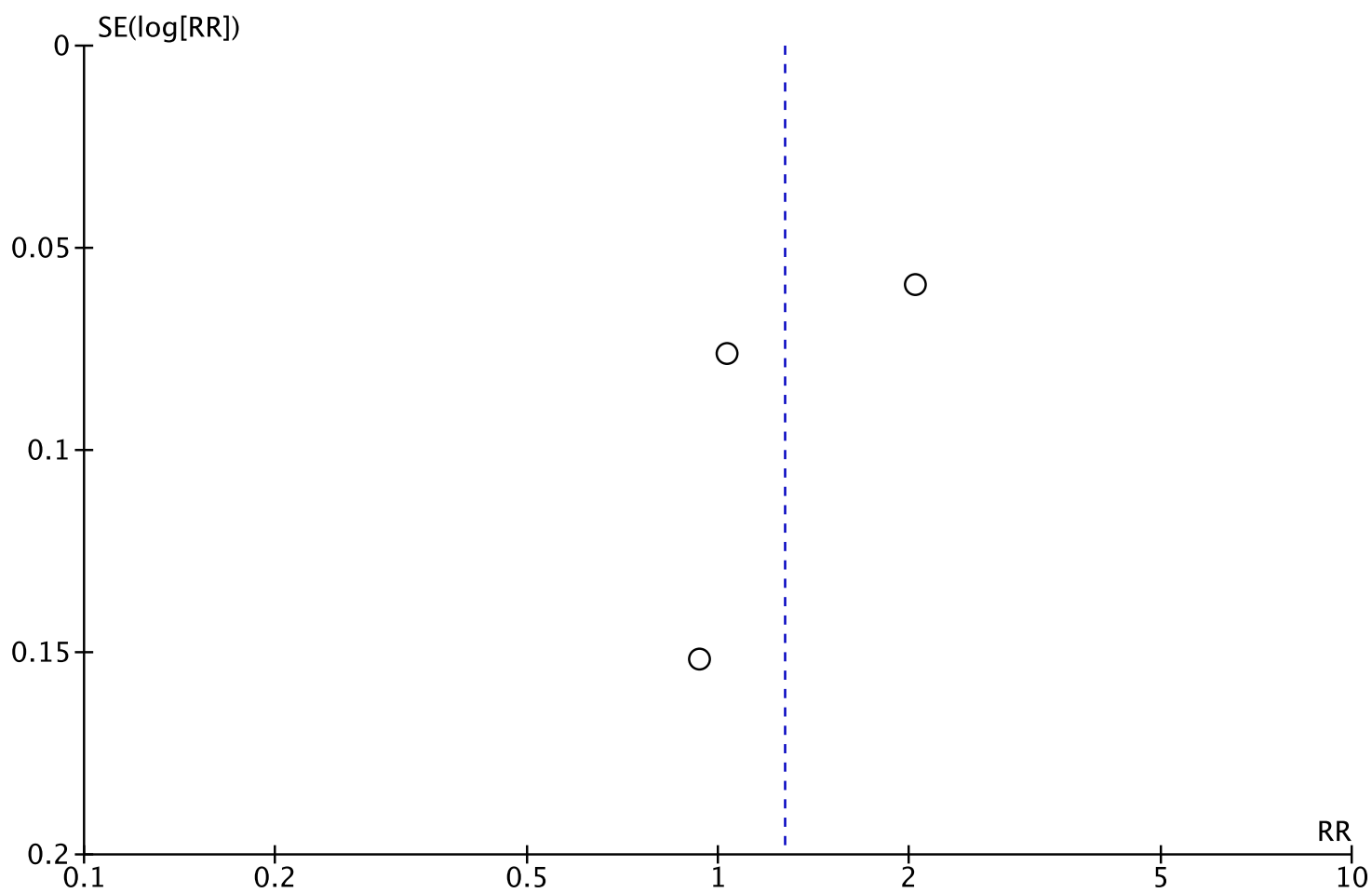

Supplement: Supplementary file 4 — Additional file 4: Fig. S3.1 Funnel plot of analysis for the association of blood group A/O and LBR, showing the results of Eggers to assess publication bias. Fig. S3.2 Funnel plot of analysis for the association of blood group B/O and LBR, showing the results of Eggers to assess publication bias. Fig. S3.3 Funnel plot of analysis for the association of blood group AB/O and LBR, showing the results of Eggers to assess publication bias. Fig. S3.4 Funnel plot of analysis for the association of blood group non-O/O and LBR, showing the results of Eggers to assess publication bias. [file 12958_2020_685_MOESM4_ESM.zip › 3.1 LBR A vs. O Funnel plot.pdf]

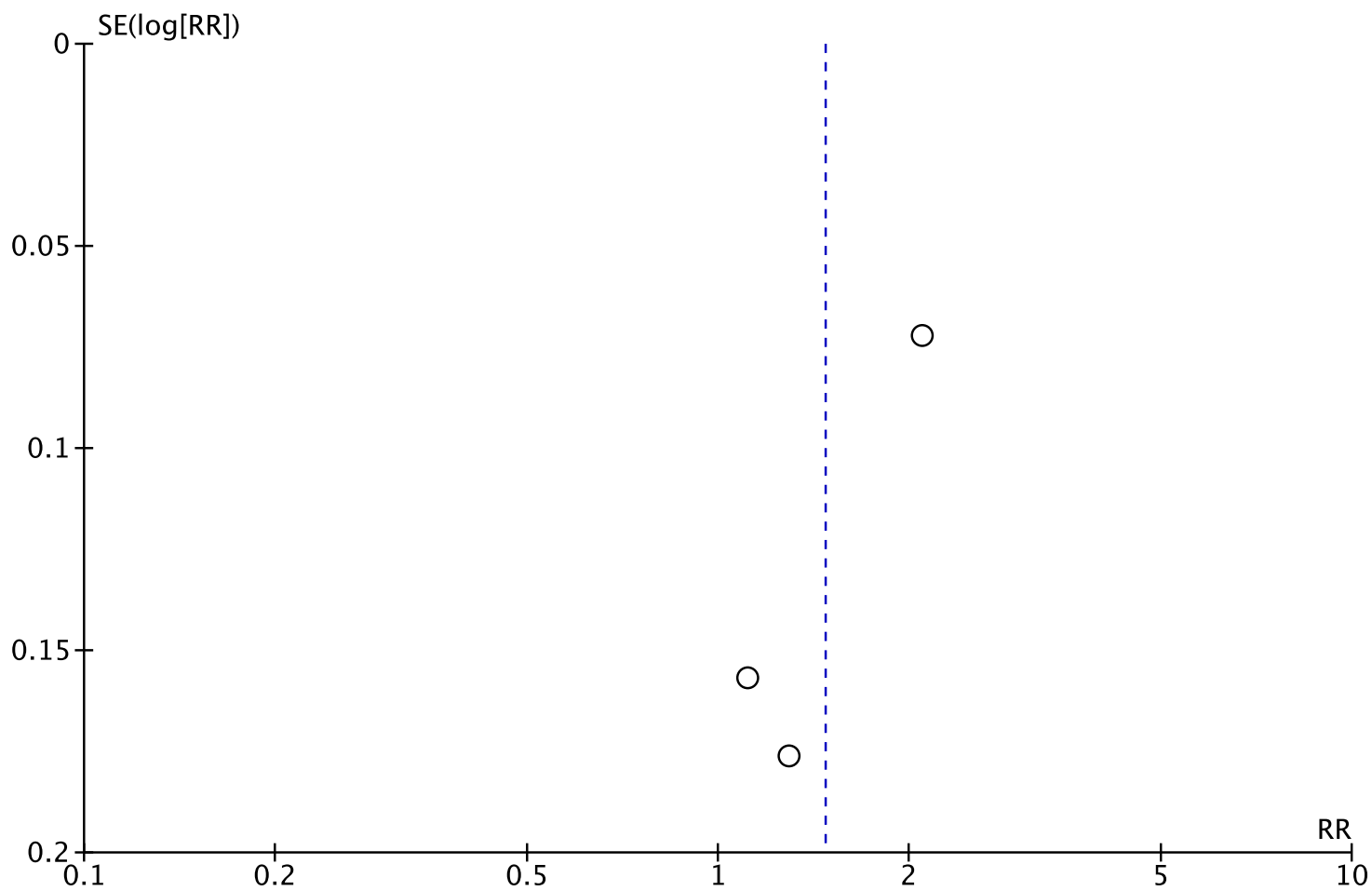

Supplement: Supplementary file 4 — Additional file 4: Fig. S3.1 Funnel plot of analysis for the association of blood group A/O and LBR, showing the results of Eggers to assess publication bias. Fig. S3.2 Funnel plot of analysis for the association of blood group B/O and LBR, showing the results of Eggers to assess publication bias. Fig. S3.3 Funnel plot of analysis for the association of blood group AB/O and LBR, showing the results of Eggers to assess publication bias. Fig. S3.4 Funnel plot of analysis for the association of blood group non-O/O and LBR, showing the results of Eggers to assess publication bias. [file 12958_2020_685_MOESM4_ESM.zip › 3.2 LBR B vs. O Funnel plot.pdf]

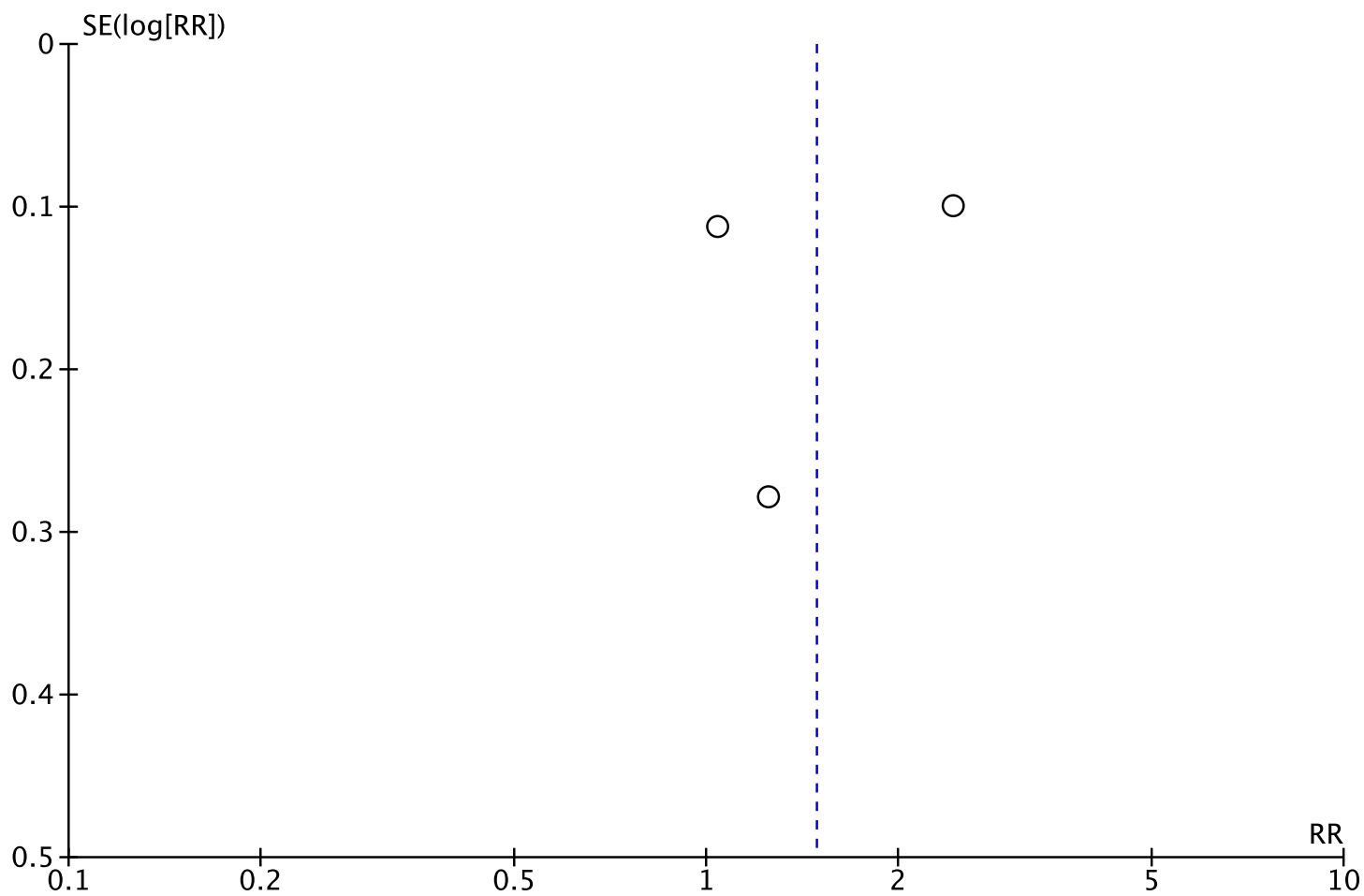

Supplement: Supplementary file 4 — Additional file 4: Fig. S3.1 Funnel plot of analysis for the association of blood group A/O and LBR, showing the results of Eggers to assess publication bias. Fig. S3.2 Funnel plot of analysis for the association of blood group B/O and LBR, showing the results of Eggers to assess publication bias. Fig. S3.3 Funnel plot of analysis for the association of blood group AB/O and LBR, showing the results of Eggers to assess publication bias. Fig. S3.4 Funnel plot of analysis for the association of blood group non-O/O and LBR, showing the results of Eggers to assess publication bias. [file 12958_2020_685_MOESM4_ESM.zip › 3.3 LBR AB vs. O Funnel plot.pdf]

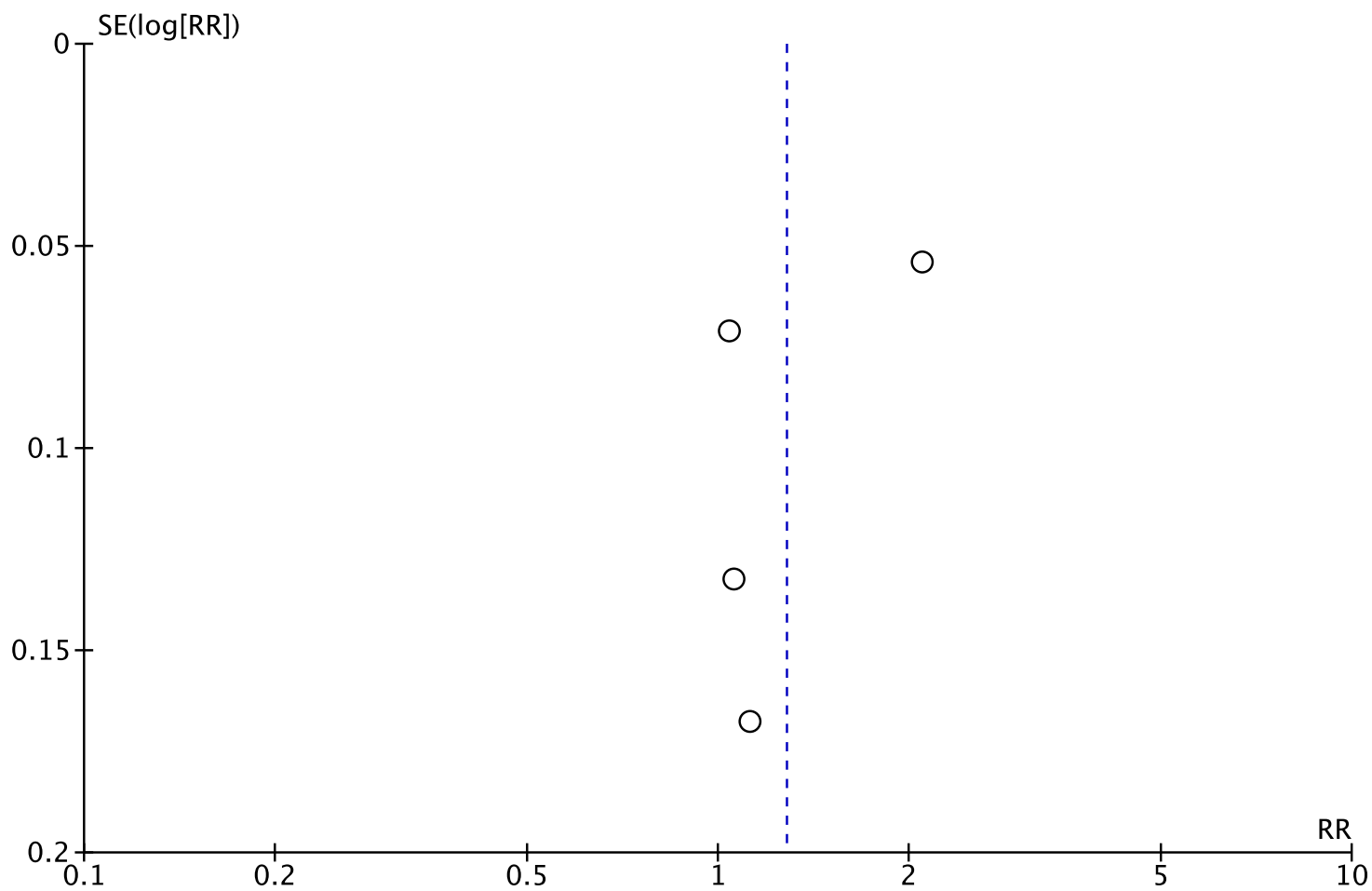

Supplement: Supplementary file 4 — Additional file 4: Fig. S3.1 Funnel plot of analysis for the association of blood group A/O and LBR, showing the results of Eggers to assess publication bias. Fig. S3.2 Funnel plot of analysis for the association of blood group B/O and LBR, showing the results of Eggers to assess publication bias. Fig. S3.3 Funnel plot of analysis for the association of blood group AB/O and LBR, showing the results of Eggers to assess publication bias. Fig. S3.4 Funnel plot of analysis for the association of blood group non-O/O and LBR, showing the results of Eggers to assess publication bias. [file 12958_2020_685_MOESM4_ESM.zip › 3.4 LBR Non-O vs. O Funnel plot.pdf]

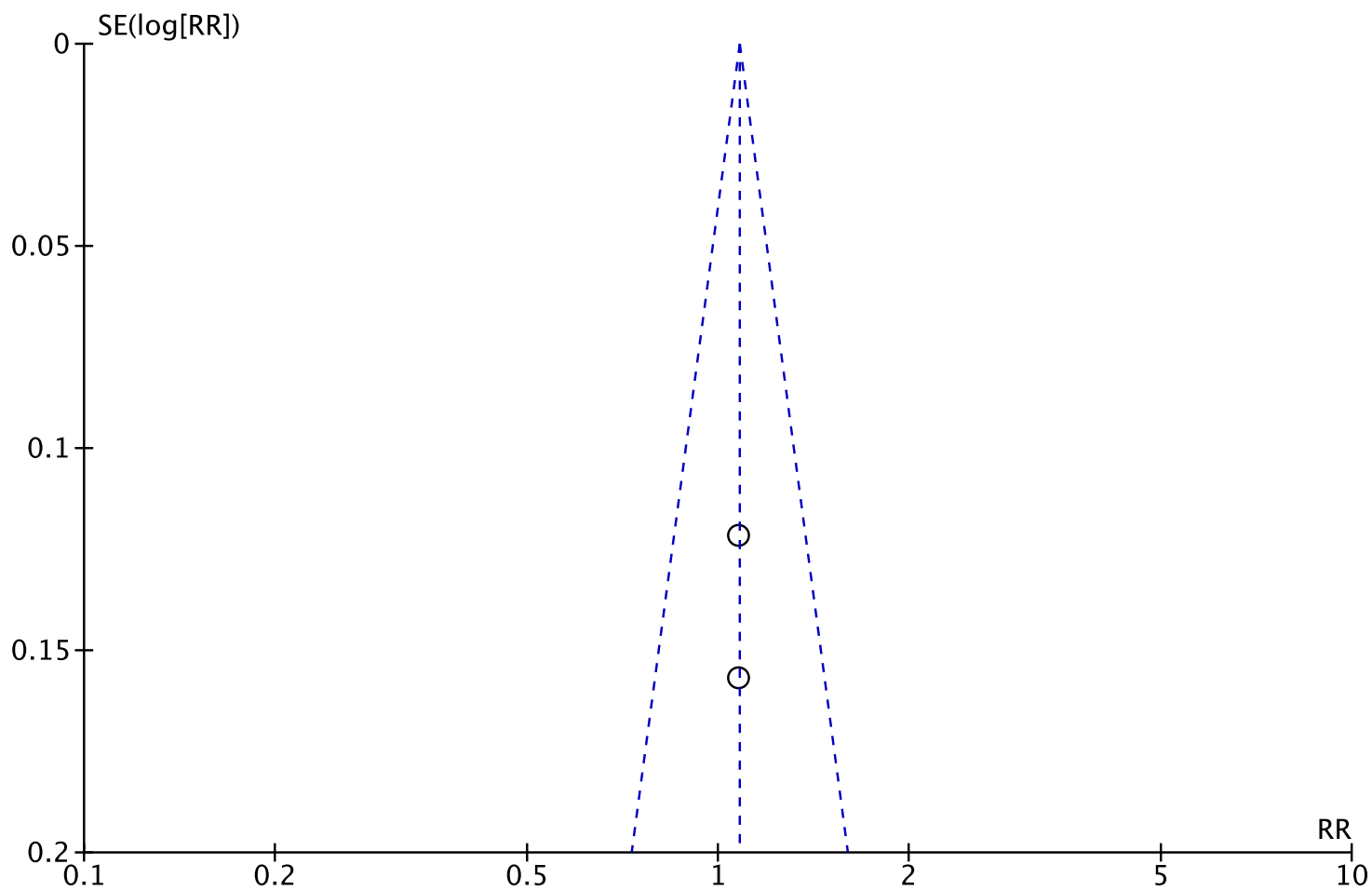

Supplement: Supplementary file 5 — Additional file 5: Fig. S4.1 Funnel plot of analysis for the association of blood group A/O and CPR, showing the results of Eggers to assess publication bias. Fig. S4.2 Funnel plot of analysis for the association of blood group B/O and CPR, showing the results of Eggers to assess publication bias. Fig. S4.3 Funnel plot of analysis for the association of blood group AB/O and CPR, showing the results of Eggers to assess publication bias. Fig. S4.4 Funnel plot of analysis for the association of blood group non-O/O and CPR, showing the results of Eggers to assess publication bias. [file 12958_2020_685_MOESM5_ESM.zip › 0715 4.2 CPR B-O Funnel plot.pdf]

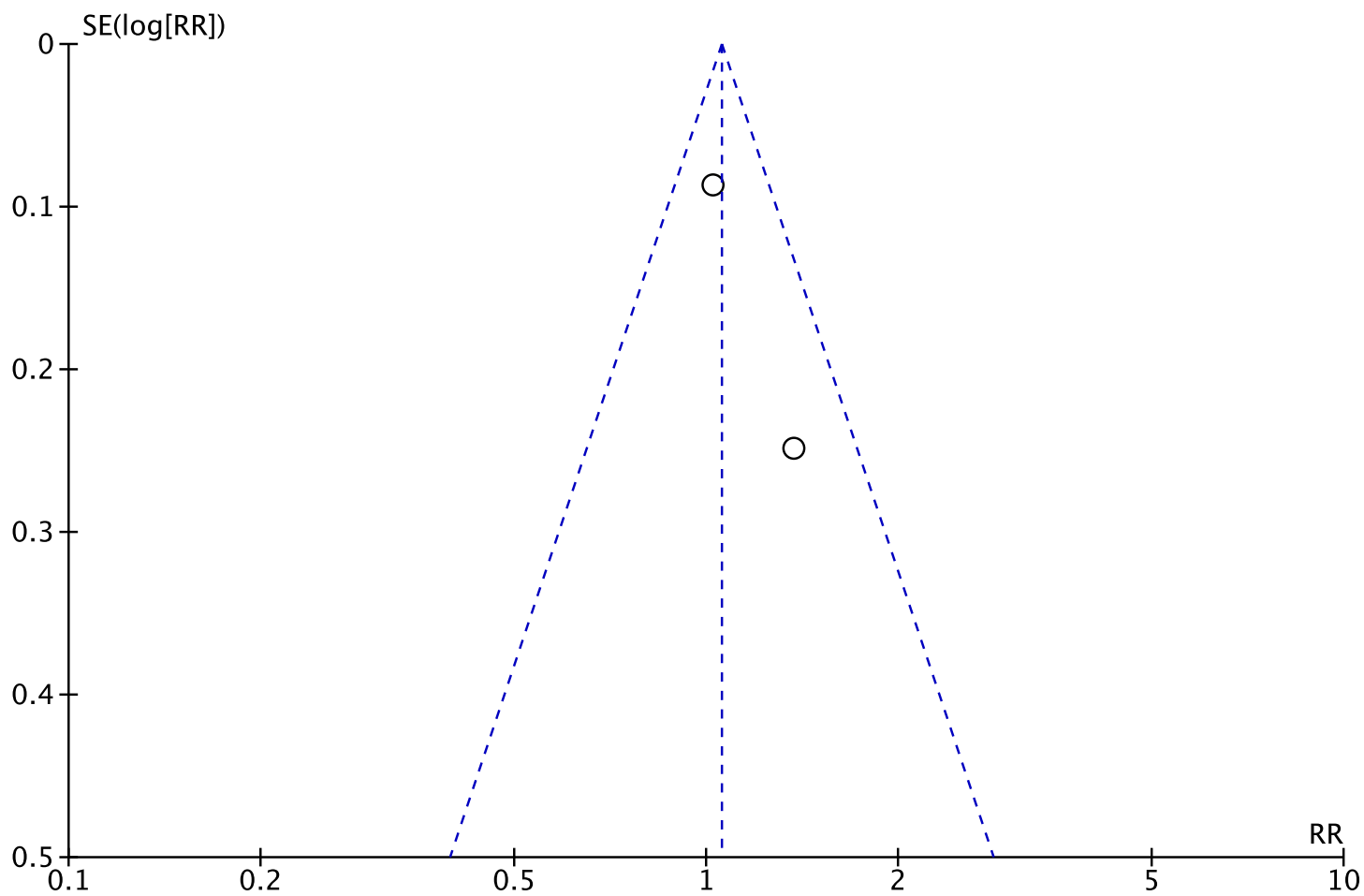

Supplement: Supplementary file 5 — Additional file 5: Fig. S4.1 Funnel plot of analysis for the association of blood group A/O and CPR, showing the results of Eggers to assess publication bias. Fig. S4.2 Funnel plot of analysis for the association of blood group B/O and CPR, showing the results of Eggers to assess publication bias. Fig. S4.3 Funnel plot of analysis for the association of blood group AB/O and CPR, showing the results of Eggers to assess publication bias. Fig. S4.4 Funnel plot of analysis for the association of blood group non-O/O and CPR, showing the results of Eggers to assess publication bias. [file 12958_2020_685_MOESM5_ESM.zip › 0715 4.3 CPR AB-O Funnel plot.pdf]

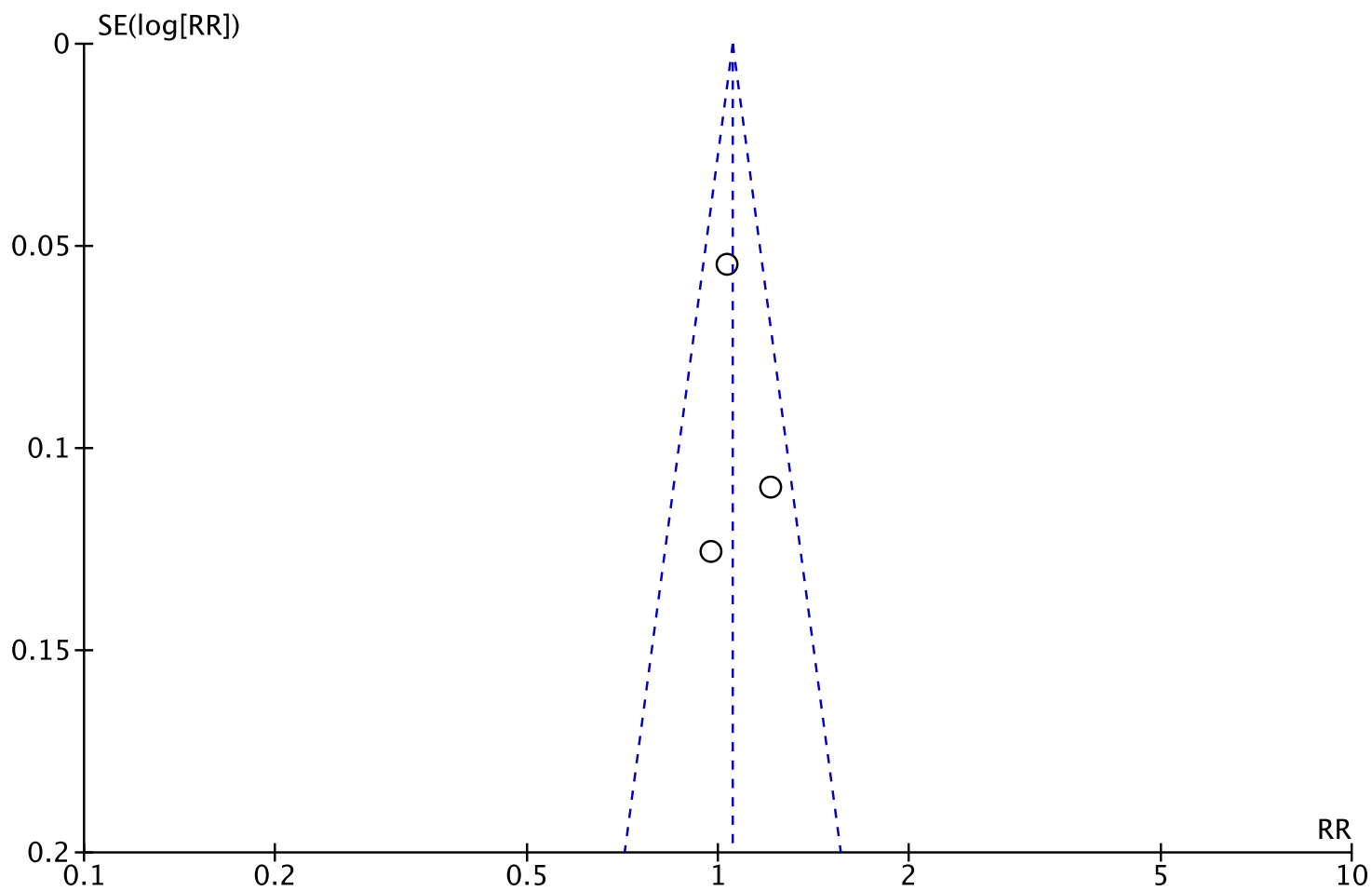

Supplement: Supplementary file 5 — Additional file 5: Fig. S4.1 Funnel plot of analysis for the association of blood group A/O and CPR, showing the results of Eggers to assess publication bias. Fig. S4.2 Funnel plot of analysis for the association of blood group B/O and CPR, showing the results of Eggers to assess publication bias. Fig. S4.3 Funnel plot of analysis for the association of blood group AB/O and CPR, showing the results of Eggers to assess publication bias. Fig. S4.4 Funnel plot of analysis for the association of blood group non-O/O and CPR, showing the results of Eggers to assess publication bias. [file 12958_2020_685_MOESM5_ESM.zip › 0715 4.4 CPR Funnel plot.pdf]

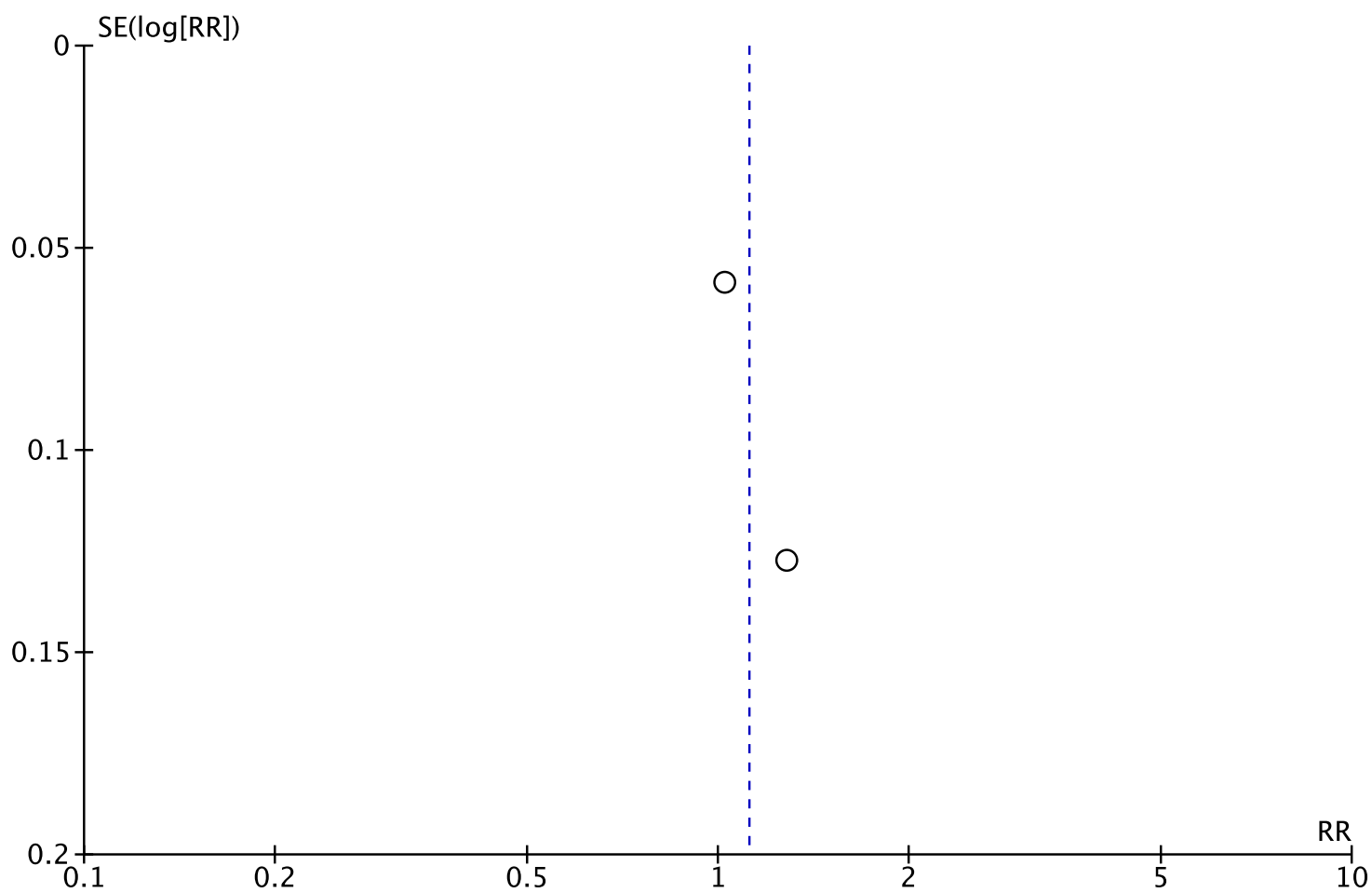

Supplement: Supplementary file 5 — Additional file 5: Fig. S4.1 Funnel plot of analysis for the association of blood group A/O and CPR, showing the results of Eggers to assess publication bias. Fig. S4.2 Funnel plot of analysis for the association of blood group B/O and CPR, showing the results of Eggers to assess publication bias. Fig. S4.3 Funnel plot of analysis for the association of blood group AB/O and CPR, showing the results of Eggers to assess publication bias. Fig. S4.4 Funnel plot of analysis for the association of blood group non-O/O and CPR, showing the results of Eggers to assess publication bias. [file 12958_2020_685_MOESM5_ESM.zip › 4.1 CPR A vs. O Funnel plot.pdf]

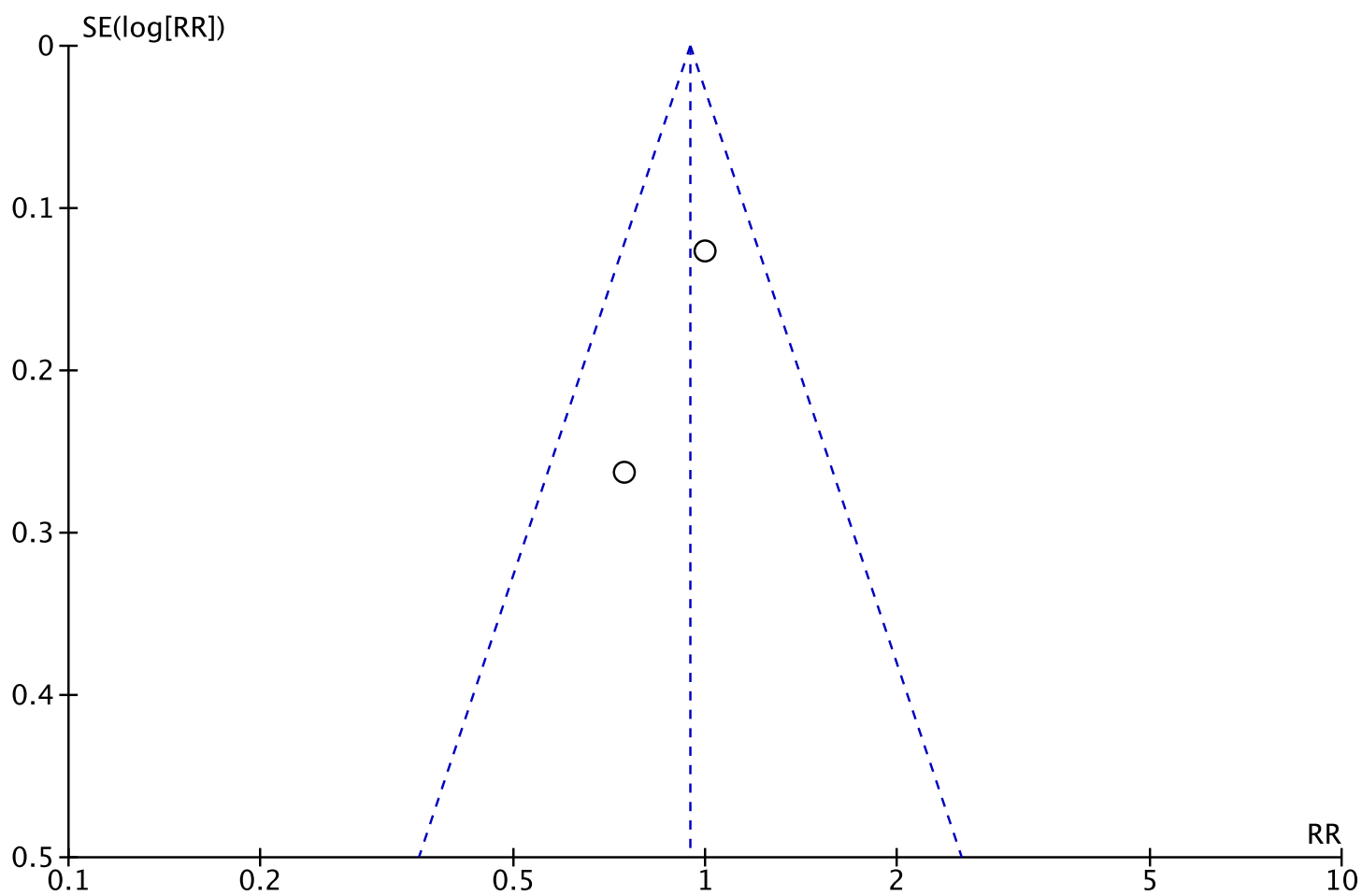

Supplement: Supplementary file 6 — Additional file 6: Fig. S5.1 Funnel plot of analysis for the association of blood group non-O/O and MR, showing the results of Eggers to assess publication bias. [file 12958_2020_685_MOESM6_ESM.pdf]
